# Disruption of *p21-activated kinase 1* gene diminishes atherosclerosis in apolipoprotein E-deficient mice

**DOI:** 10.1038/ncomms8450

**Published:** 2015-06-24

**Authors:** Nikhlesh K. Singh, Sivareddy Kotla, Elena Dyukova, James G. Traylor Jr., A. Wayne Orr, Jonathan Chernoff, Tony N. Marion, Gadiparthi N. Rao

**Affiliations:** 1 Department of Physiology, University of Tennessee Health Science Center, 894 Union Avenue, Memphis, Tennessee 38163, USA; 2 Department of Pathology, LSU Health Sciences Center, Shreveport, Louisiana 71103, USA; 3 Fox Chase Cancer Center, 333 Cottman Avenue, Philadelphia, Pennsylvania 19111, USA; 4 Department of Microbiology, Immunology and Biochemistry, University of Tennessee Health Science Center, Memphis, Tennessee 38163, USA

## Abstract

Pak1 plays an important role in various cellular processes, including cell motility, polarity, survival and proliferation. To date, its role in atherogenesis has not been explored. Here we report the effect of Pak1 on atherogenesis using atherosclerosis-prone apolipoprotein E-deficient (ApoE^−/−^) mice as a model. Disruption of Pak1 in ApoE^−/−^ mice results in reduced plaque burden, significantly attenuates circulating IL-6 and MCP-1 levels, limits the expression of adhesion molecules and diminishes the macrophage content in the aortic root of ApoE^−/−^ mice. We also observed reduced oxidized LDL uptake and increased cholesterol efflux by macrophages and smooth muscle cells of ApoE^−/−^:Pak1^−/−^ mice as compared with ApoE^−/−^ mice. In addition, we detect increased Pak1 phosphorylation in human atherosclerotic arteries, suggesting its role in human atherogenesis. Altogether, these results identify Pak1 as an important factor in the initiation and progression of atherogenesis.

## Abstract (web summary)

Atherogenesis involves coordinated action of different cell types and factors. Here the authors show that the kinase Pak1 represents a key pro-atherogenic factor affecting the function of macrophages and vascular smooth muscle cells, including their production of proinflammatory cytokine IL-6 and chemokine MCP-1, and retention of cholesterol.

## Graphical abstract

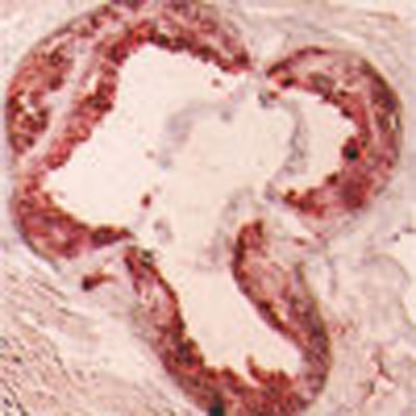

Atherosclerosis, a disease of large arteries, is the foremost cause of heart disease and stroke, and is the leading cause of death and disability in the industrialized countries^1^. Atherosclerotic lesions involve a series of cellular and molecular changes^2^. Endothelial dysfunction, which represents the earliest changes during atherosclerotic lesion formation, results in an increase in lipid permeability of the endothelium, macrophage recruitment, foam-cell formation and homing of T lymphocytes and platelets^3^. The intimal lipid accumulation and local inflammation are closely linked to the pathogenesis of atherosclerosis and macrophages appear to play a central role in the deregulation of vessel-wall lipid homeostasis and orchestrating inflammatory responses^2^. The dysfunctional endothelium and the infiltrated inflammatory cells by releasing a plethora of growth factors and cytokines may exert phenotypic changes in vascular smooth muscle cells (VSMCs), converting them from quiescent contractile state to the synthetic active state^4^. Therefore, proliferation and migration of VSMCs along with their apoptosis may also play a role in the pathogenesis of atherosclerosis^5^.

p21-Activated kinases (Paks), a family of serine/threonine kinases, are the effector molecules of the small GTPases Cdc42 and Rac^6^. Paks play crucial roles in various cellular processes, including cell motility, polarity, survival and proliferation^7^. Most of the eukaryotes encode one or more *Pak* gene, which underlies an important physiological function for this family of kinases^6^,^7^. In fact, the recent development in generating Pak knockout animal models, in particular mouse and zebrafish models, has revealed their essential role in the development and function of various organ systems, including heart and blood vessels^8^,^9^,^10^,^11^,^12^. In addition to their role in the development and functions of various cellular systems, some studies have shown that Paks play a role in disease processes such as cancer^13^. It has also been demonstrated that activation of Pak1 might play a role in cholesterol trafficking, as it mediates the downregulation of the scavenger receptor B1 promoter activity^14^. In addition, some studies have suggested that Pak plays a role in paracellular pore formation and thereby in the regulation of vascular permeability in response to cellular and atherogenic stimuli^15^,^16^. In recent times, a study has demonstrated that Pak1 mediates activation of inflammatory signalling events in endothelial cells^17^. The latter observations may infer that Pak1 might be playing a role in the pathogenesis of atherosclerosis, but to date the contribution of Pak1 in atherogenesis is unknown.

Here we found that apolipoprotein E-deficient (ApoE^−/−^) mice have elevated levels of Pak1 and its activity in the aorta and macrophages. Based on this finding, we hypothesized that Pak1 plays a central role in atherogenesis. To test this hypothesis, we crossed Pak1 knockout mice to atherosclerosis-prone ApoE^−/−^ mice, to generate ApoE^−/−^:Pak1^−/−^ mice. The ApoE^−/−^ and ApoE^−/−^:Pak1^−/−^ mice were then fed with Western diet (WD) for 16 weeks, starting from 8 weeks of age, and atherosclerosis was assessed by evaluating the lesion size and its composition in the aorta and aortic root. Knocking out Pak1 caused significant decrease in circulating interleukin (IL)-6 and monocyte chemoattractant protein (MCP)-1 levels, diminished aortic root macrophage content, decreased cluster of differentiation 36 (CD36) levels with reduced oxidized low-density lipoprotein (OxLDL) uptake and increased ABCA1 levels with enhanced cholesterol efflux by both peritoneal macrophages and VSMCs, leading to a reduction in plaque burden in ApoE^−/−^ mice. Together, these findings demonstrate that Pak1 plays a crucial role in the pathogenesis of atherosclerosis.

## Results

### Increased Pak1 expression and its activity in ApoE^−/−^ mice

**Figure 1:**
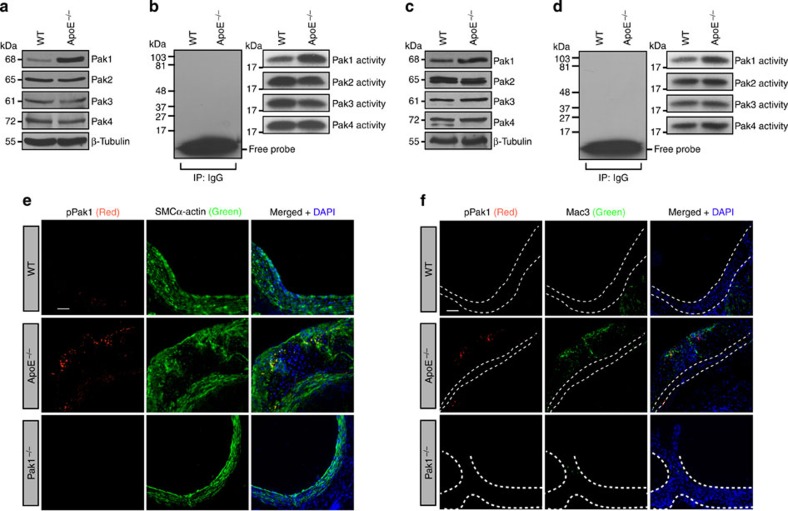
Pak1 expression and its activity increase in ApoE^−/−^ mice. Aortic or primary peritoneal macrophage extracts of 24-week-old C57BL/6 (WT) and ApoE^−/−^ mice fed with CD were analysed either by western blotting for the indicated molecules using their specific antibodies (**a**,**c**) or an equal amount of protein from WT and ApoE^−/−^ mice were immunoprecipated (IP) with rabbit IgG or anti-Pak1-4 antibodies and the immunocomplexes were assayed for kinase activity using MBP and [γ-^32^P]-ATP as substrates (**b**,**d**). (**e**,**f**) Aortic root cryosections of WT, ApoE^−/−^ and Pak1^−/−^ mice were stained by double immunofluorescence for pPak1 (red) and SMCα-actin or Mac3 (green). Scale bar, 50 μm (**e**,**f**).

Paks are known to phosphorylate a wide range of substrates, many of which might be relevant to the heart and vasculature development and function^10^,^11^,^12^. In recent times, it was shown that Pak mediates paracellular pore formation and thereby vascular endothelial permeability to a wide range of cellular and atherogenic stimulants^15^,^16^. To test the role of Paks in atherogenesis, we analysed Pak1, Pak2, Pak3 and Pak4 levels, and their activities in the aorta and peritoneal macrophages of 24-week-old female and male ApoE^−/−^ and C57BL/6 (wild type (WT)) mice. We found an increase in Pak1 expression and its activity in the aorta of ApoE^−/−^ mice as compared with that in WT mice (Fig. 1a,b). We chose to study 24-week-old ApoE^−/−^ mice, because these mice have been shown to develop advanced atherosclerotic lesions after 15 weeks of age^18^. We have not observed any change in Pak2, Pak3 or Pak4 levels, or their activities in the aorta of ApoE^−/−^ mice as compared with that in WT mice. Electron microscopic analysis of fibrous plaques from ApoE^−/−^ mice have revealed the presence of large, lipid-laden macrophage-derived foam cells^18^. Therefore, we also measured Pak1, Pak2, Pak3 and Pak4 levels, and their activities in the peritoneal macrophages of ApoE^−/−^ and WT mice. A significant increase in the expression and activity of Pak1 was found in the peritoneal macrophages of ApoE^−/−^ mice as compared with that in WT mice (Fig. 1c,d). To relate the above findings on the increased expression and activity of Pak1 in the aorta and peritoneal macrophages to atherosclerotic lesions, we examined Pak1 phosphorylation (Thr423) in the aortic roots of ApoE^−/−^ and WT mice in combination with SMC and macrophage-specific markers. Immunoflluorescence staining of the aortic root sections showed increased co-localization of pPak1 with SMCα-actin and Mac3 in ApoE^−/−^ mice as compared with WT mice (Fig. 1e,f). To confirm the specificity of pPak1 immunostaining, we used aorta from Pak1^−/−^ mice as a negative control. No staining was observed with anti-pPak1 antibody (Ab) in either SMC or macrophages, which confirms the specificity of the immunostaining (Fig. 1e,f).

### Pak1 deficiency diminishes atherosclerosis in ApoE^−/−^ mice

**Figure 2:**
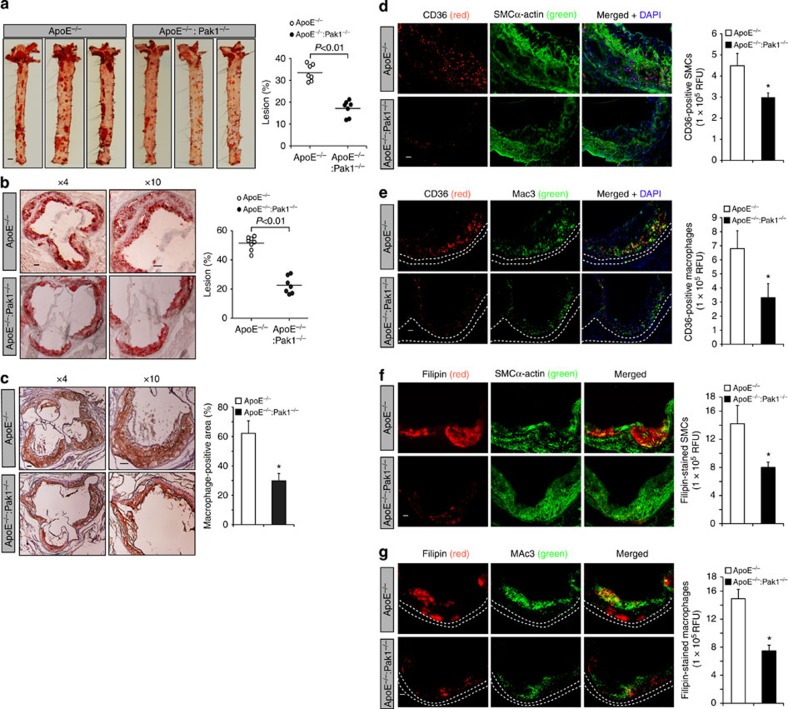
Deletion of Pak1 reduces atherosclerotic plaque progression. (**a**) Representative *en face* staining of aortas from ApoE^−/−^ and ApoE^−/−^:Pak1^−/−^ mice fed with WD for 16 weeks are shown and the plaque area is presented as lesion % (that is, % of whole aorta) in the graph. Each symbol represents one animal and the horizontal bar indicates the mean value. (**b**) Representative Oil red O staining of the aortic root sections of the mice described in **a** is shown and the graph represents the quantification of the area positive for lipid staining. (**c**) Aortic root sections of the mice described in **a** stained for Mac3 are shown and the graph represents the quantification of the area positive for macrophages. Aortic root cryosections of the mice described in **a** were analysed by double immunofluorescence staining for CD36 in combination with SMCα-actin or Mac3 (**d**,**e**), or Filipin in combination with SMCα-actin or Mac3 (**f**,**g**). Bar graph represents the quantification of aortic root sections for CD36 and Filipin staining (*n*=6). Data were presented as mean±s.d. and assessed by Student’s *t*-test. *P<0.05 versus ApoE^−/−^ mice. Scale bars, 1 mm (**a**), 100 μm (**b**,**c**) and 50 μm (**d**–**g**).

To study the role of Pak1 in atherogenesis, we fed male ApoE^−/−^ and ApoE^−/−^:Pak1^−/−^ mice with WD starting at 8 weeks of age for 16 weeks and analysed them for atheroma formation. Although no differences were found in the body weight between ApoE^−/−^ and ApoE^−/−^:Pak1^−/−^ mice, ApoE^−/−^:Pak1^−/−^ mice showed lower levels of serum cholesterol, LDL and triglycerides (TGs) as compared with ApoE^−/−^ mice (Table 1). Measurement of total atherosclerotic lesion area by *en face* lipid staining revealed a *∼*51% decrease in the aortic lesions of ApoE^−/−^ mice lacking Pak1 (17.14%±3.64 in ApoE^−/−^:Pak1^−/−^ versus 33.48±3.778 in ApoE^−/−^ mice; *P*<0.01 by Student’s *t*-test; Fig. 2a). The percentage of plaque area in the aortic roots of WD-fed ApoE^−/−^:Pak1^−/−^ mice was decreased by ∼44% as compared with those in ApoE^−/−^ mice (Fig. 2b). The macrophage content was also found decreased in the aortic roots of ApoE^−/−^:Pak1^−/−^ mice as compared with that in ApoE^−/−^ mice (Fig. 2c). CD36, also known as FAT (fatty acid translocase), was identified as a key receptor for OxLDL in both *in-vitro* and *in-vivo* studies, contributing to foam-cell formation and atherogenesis^19^,^20^,^21^. Hence, to investigate the role of Pak1 on CD36 expression, we compared CD36 levels in the aortic root plaque area of ApoE^−/−^ and ApoE^−/−^:Pak1^−/−^ mice. A decrease in CD36 expression was observed both in SMCs and macrophages embedded in the plaque areas of ApoE^−/−^:Pak1^−/−^ mice as compared with that in ApoE^−/−^ mice (Fig. 2d,e). In addition, we found a decrease in cholesterol deposition both in SMCs and macrophages of the plaque areas of ApoE^−/−^:Pak1^−/−^ mice as compared with that in ApoE^−/−^ mice (Fig. 2f,g)

**Figure 3:**
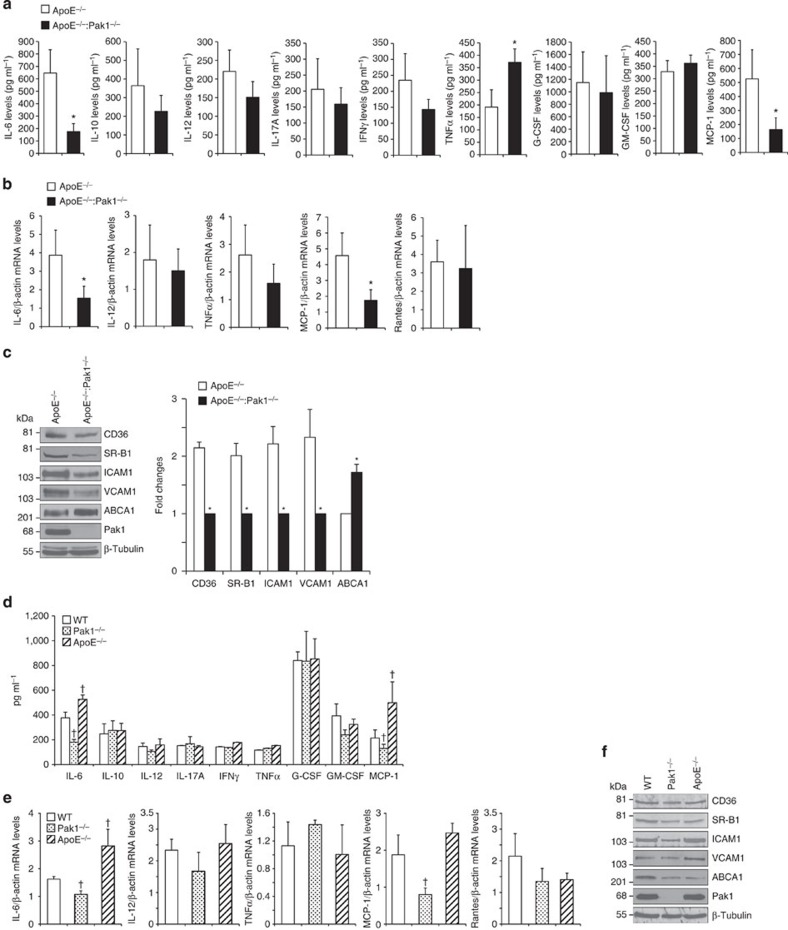
Lack of Pak1 downregulates IL-6 and MCP-1 levels in ApoE^−/−^ mice. (**a**) The plasma from ApoE^−/−^ and ApoE^−/−^:Pak1^−/−^ mice fed with WD for 16 weeks were analysed for the indicated inflammatory cytokines and MCP-1 levels. (**b**) RNA was isolated from aortic arch region of ApoE^−/−^ and ApoE^−/−^:Pak1^−/−^ mice fed with WD for 16 weeks and qRT–PCR analysis for the indicated cytokines and chemokines was performed. (**c**) An equal amount of protein from the aortic extracts of the mice described in **a** was analysed by western blotting for the indicated proteins using their specific antibodies. Bar graph in **c** represents the quantification of three western blottings each from a group of two pooled arteries. (**d**) The plasma from WT, Pak1^−/−^ and ApoE^−/−^ mice fed with CD were analysed for the indicated inflammatory cytokines and MCP-1 levels. (**e**) RNA was isolated from aortic arch region of the mice described in **d** and qRT–PCR analysis was performed for the indicated cytokines and chemokines. (**f**) An equal amount of protein from the aortic extracts of the mice described in **d** was analysed by western blotting for the indicated proteins using their specific antibodies. Data were presented as mean±s.d. and assessed by Student’s *t*-test. *P<0.01 versus ApoE^−/−^ mice (*n*=6 mice); †*P*<0.05 versus WT mice (*n*=6).

To examine the mechanisms of reduced atherogenesis in ApoE^−/−^:Pak1^−/−^ mice, we measured the plasma levels of inflammatory cytokines such as IL-6, IL-10, IL-12, IL-17A, interferon-γ, tumour necrosis factor (TNF)-α, granulocyte colony-stimulating factor and granulocyte–macrophage colony-stimulating factor, and chemokine MCP-1 in ApoE^−/−^ and ApoE^−/−^:Pak1^−/−^ mice. ApoE^−/−^:Pak1^−/−^ mice showed a 50% decrease in circulating IL-6 and MCP-1 levels as compared with ApoE^−/−^ mice (Fig. 3a). In contrast, a 40% increase in TNFα levels was observed in ApoE^−/−^:Pak1^−/−^ mice as compared with that in ApoE^−/−^ mice (Fig. 3a). No significant differences were observed in the levels of IL-10, IL-12, IL-17A, interferon-γ, granulocyte colony-stimulating factor and granulocyte–macrophage colony-stimulating factor between ApoE^−/−^ and ApoE^−/−^:Pak1^−/−^ mice. Quantitative reverse transcriptase–PCR (qRT–PCR) analysis of RNA isolated from the aortic arch regions showed a significant decrease for IL-6 and MCP-1 messenger RNA levels in ApoE^−/−^:Pak1^−/−^ mice as compared with that in ApoE^−/−^ mice, thus correlating with their reduced plasma levels (Fig. 3b). In regard to TNFα levels, despite its increased plasma levels in ApoE^−/−^:Pak1^−/−^ mice, no significant difference was noted in its mRNA levels in the aortic arch regions of ApoE^−/−^ and ApoE^−/−^:Pak1^−/−^ mice (Fig. 3b). No major differences were observed in IL-12 and Rantes mRNA levels. A substantial body of evidence suggests that IL-6 and MCP-1 play a role in enhancing atherosclerotic lesion formation and plaque progression^22^,^23^. Furthermore, a study conducted on human subjects revealed an association of IL-6 with subclinical atherosclerotic lesions and its correlation with ICAM1 expression^24^. Therefore, to find a link, if any, between Pak1 and adhesion molecules, Pak1 and scavenger receptors or Pak1 and reverse cholesterol transporters, we isolated the aortic arch regions from ApoE^−/−^ and ApoE^−/−^:Pak1^−/−^ mice fed with WD for 16 weeks, prepared tissue extracts and analysed for ICAM1, VCAM1, CD36, SR-B1 and ABCA1 expression. We observed a decrease in the expression levels of ICAM1, VCAM1, CD36 and SR-B1 in ApoE^−/−^:Pak1^−/−^ mice as compared with that in ApoE^−/−^ mice (Fig. 3c). In contrast, the levels of ABCA1 were increased in aortic arch regions of ApoE^−/−^:Pak1^−/−^ mice as compared with that of ApoE^−/−^ mice (Fig. 3c), and this finding correlated with reduced plaque burden in ApoE^−/−^:Pak1^−/−^ mice, as ABCA1 mediates cholesterol efflux to high-density lipoprotein (HDL), and thereby prevent foam cell formation^25^. To find whether the observed changes in the above-listed molecules in ApoE^−/−^:Pak1^−/−^ mice compared with ApoE^−/−^ mice were due to an indirect consequence of decreased plasma LDL or smaller plaques, we measured the plasma levels of cytokines and chemokine MCP-1 in 6-week-old WT, Pak1^−/−^ and ApoE^−/−^ mice that were maintained on chow diet (CD). Among the molecules measured, we observed a significant decrease in the circulating IL-6 and MCP-1 levels in CD-fed Pak1^−/−^ mice as compared with that in WT mice (Fig. 3d). qRT–PCR analysis also showed decreased mRNA levels of IL-6 and MCP-1 in the aortic arch regions of CD-fed Pak1^−/−^ mice as compared with that of WT mice (Fig. 3e). In regard to their levels in ApoE^−/−^ mice, both IL-6 and MCP-1 levels were increased in the plasma and aorta of these mice as compared with that in WT mice (Fig. 3d,e). No significant changes were observed in CD36, SR-B1, ICAM1, VCAM1 and ABCA1 levels in the aortic arch regions of CD-fed WT, Pak1^−/−^ or ApoE^−/−^ mice (Fig. 3f). To further characterize the Pak^−/−^ phenotype, we also measured the plasma lipid levels. Although no major differences were observed in the plasma lipid levels between WT and Pak^−/−^ mice, the cholesterol, HDL, LDL and TG levels were found to be higher in ApoE^−/−^ and ApoE^−/−^:Pak1^−/−^ mice as compared with that in WT or Pak1^−/−^ mice that were maintained on CD (Table 2). These results indicate that Pak1 deficiency attains the phenotypic characteristics of decreased IL-6 and MCP-1 without having much effect on cholesterol homeostasis. In contrast, the lack of ApoE^−/−^ causes an elevation in the plasma cholesterol, HDL, LDL and TG levels irrespective of Pak1 deficiencey (Table 2).

### Pak1 regulates macrophage content in aortas of ApoE^−/−^ mice

**Figure 4:**
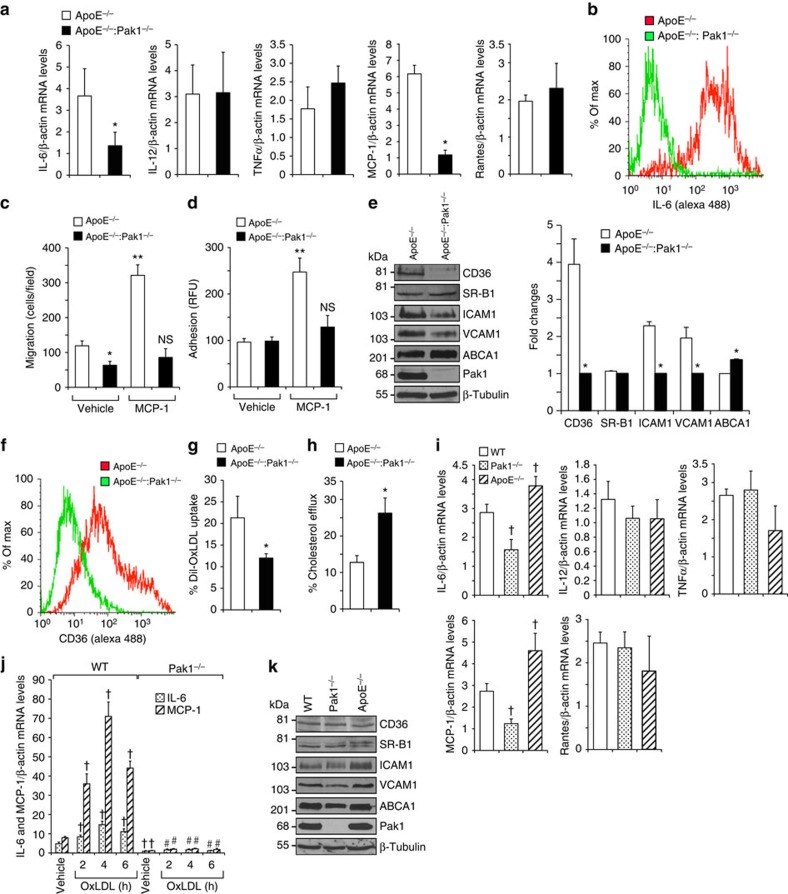
Pak1 deficiency affects macrophage function. (**a**) RNA from peritoneal macrophages of ApoE^−/−^ and ApoE^−/−^:Pak1^−/−^ mice fed with WD for 16 weeks was analysed by qRT–PCR for the indicated cytokines and chemokines. (**b**) FACS analysis of IL-6-positive peritoneal macrophages from ApoE^−/−^ and ApoE^−/−^:Pak1^−/−^ mice fed with WD for 16 weeks is shown. Peritoneal macrophages isolated from the mice described in **a** were subjected to MCP-1 (50 ng ml^−1^)-induced migration (**c**) and adhesion (**d**). (**e**) Extracts of peritoneal macrophages isolated from the mice described in **a** were analysed by western blotting for the indicated proteins using their specific antibodies. Bar graph in **e** represents the quantification of three western blottings each from a group of two pooled arteries. (**f**) FACS analysis of CD36-positive peritoneal macrophages isolated from the mice described in **a** is shown. Peritoneal macrophages from ApoE^−/−^ and ApoE^−/−^:Pak1^−/−^ mice fed with WD for 16 weeks were analysed for Dil-OxLDL uptake (**g**) and cholesterol efflux (**h**). (**i**) RNA from peritoneal macrophages of WT, Pak1^−/−^ and ApoE^−/−^ mice fed with CD was analysed by qRT–PCR for the indicated cytokines and chemokines. (**j**) Peritoneal macrophages of WT and Pak1^−/−^ mice fed with CD were treated with OxLDL (10 μg ml^−1^) for the indicated time periods and RNA was isolated and analysed by qRT–PCR for IL-6 and MCP-1 levels. (**k**) Extracts of peritoneal macrophages isolated from the mice described in **i** were analysed by western blotting for the indicated proteins using their specific antibodies. Data were presented as mean±s.d. and assessed by Student’s *t*-test. *P<0.01 versus ApoE^−/−^ mice (*n*=6); **P<0.01 versus vehicle control; †*P*<0.01 versus WT mice (*n*=6) or WT+vehicle, #*P*<0.01 versus WT mice*+*OxLDL (*n*=6). NS, not significant compared with vehicle control.

To asses whether ApoE^−/−^:Pak1^−/−^ macrophages have a defect in cytokine and chemokine production, we analysed the mRNA levels of IL-6, IL-12, TNFα, MCP-1 and Rantes by qRT–PCR in the peritoneal macrophages of ApoE^−/−^ and ApoE^−/−^:Pak1^−/−^ mice fed with WD for 16 weeks. Although no differences were found in the levels of IL-12, TNFα and Rantes, a significant decrease was observed in IL-6 and MCP-1 mRNA levels in the macrophages of ApoE^−/−^:Pak1^−/−^ mice as compared with that in ApoE^−/−^ mice (Fig. 4a). The flow cytometry analysis also showed a decrease in IL-6-positive macrophages in ApoE^−/−^:Pak1^−/−^ mice as compared with ApoE^−/−^ mice (Fig. 4b). MCP-1 promotes monocyte recruitment to the aorta and its differentiation into macrophages^26^. Therefore, we examined the effect of MCP-1 on the migration of peritoneal macrophages isolated from ApoE^−/−^ and ApoE^−/−^: Pak1^−/−^ mice. A significant decrease was observed in the migration capacity of macrophages isolated from ApoE^−/−^:Pak1^−/−^ mice as compared with those from ApoE^−/−^ mice and the lack of Pak1 not only blocked the basal but also attenuated MCP-1-induced migration of these cells (Fig. 4c). We also performed *in-vitro* macrophage adhesion assay, in which we measured the capacity of peritoneal macrophages of ApoE^−/−^ and ApoE^−/−^:Pak1^−/−^ mice to adhere to a monolayer of mouse pancreatic endothelial cells. A decrease in ApoE^−/−^:Pak1^−/−^ macrophage adhesion to endothelial monolayer was observed in response to MCP-1 as compared with ApoE^−/−^ mice (Fig. 4d). These observations may suggest that besides downregulation of MCP-1, the Pak1 deficiency may also affect the MCP-1-induced chemotactic signalling, perhaps due to its role in cytoskeleton remodelling. To understand the possible mechanisms underlying the Pak1 role in the regulation of monocyte migration/adhesion, we analysed the expression levels of ICAM1 and VCAM1 in the peritoneal macrophages of ApoE^−/−^ and ApoE^−/−^:Pak1^−/−^ mice. Decreased expression levels of ICAM1 and VCAM1 were found in macrophages of ApoE^−/−^:Pak1^−/−^ mice as compared with that in ApoE^−/−^ mice (Fig. 4e). To examine the potential function of Pak1 in macrophage lipid deposition, we also measured the levels of CD36, SR-B1 and ABCA1 in the peritoneal macrophages of both ApoE^−/−^ and ApoE^−/−^:Pak1^−/−^ mice. Although CD36 levels were decreased, ABCA1 levels were increased in the peritoneal macrophages of ApoE^−/−^:Pak1^−/−^ mice as compared with ApoE^−/−^ mice (Fig. 4e). In regard to SR-B1 expression, no major differences were observed in its levels in macrophages of ApoE^−/−^:Pak1^−/−^ mice as compared with ApoE^−/−^ mice (Fig. 4e). Consistent with CD36 expression levels, a decrease in CD36-positive macrophages along with a decreased ability of Dil-OxLDL uptake was found in ApoE^−/−^:Pak1^−/−^ mice as compared with ApoE^−/−^ mice (Fig. 4f,g), suggesting a possible role of Pak1 in OxLDL uptake as well. In contrast, a 50% increase in cholesterol efflux was observed in ApoE^−/−^:Pak1^−/−^ macrophages as compared with ApoE^−/−^ macrophages (Fig. 4h) and these findings correlated with ABCA1 levels. These results indicate that genetic elimination of Pak1 significantly decreases the intracellular cholesterol accumulation in macrophages. To ascertain the role of Pak1 in the modulation of IL-6 and MCP-1 in macrophages, we measured the levels of cytokines, chemokines, adhesion molecules, scavenger receptors and reverse cholesterol transporters in the macrophages of 6-week-old WT, Pak1^−/−^ and ApoE^−/−^ mice that were maintained on CD. Although there were no significant changes in the levels of IL-12, TNFα and Rantes, both IL-6 and MCP-1 levels were reduced in the macrophages of Pak1^−/−^ mice as compared with that in WT mice (Fig. 4i). To further test the role of Pak1 on IL-6 and MCP-1 expression and atherogenesis, we isolated peritoneal macrophages from CD-fed WT and Pak1^−/−^ mice, treated with and without atherogenic stimulant OxLDL for various time periods, and IL-6 and MCP-1 levels were measured. OxLDL induced IL-6 and MCP-1 expression in the peritoneal macrophages of WT but not Pak1^−/−^ mice (Fig. 4j). Little or no changes were observed in the levels of adhesion molecules, scavenger receptors and reverse cholesterol transporters in the peritoneal macrophages of WT, Pak1^−/−^ and ApoE^−/−^ mice (Fig. 4k). These observations further reinforce that Pak1 regulates IL-6 and MCP-1 even in macrophages.

### Pak1 regulates VSMC migration and fibrofatty lesion formation

**Figure 5:**
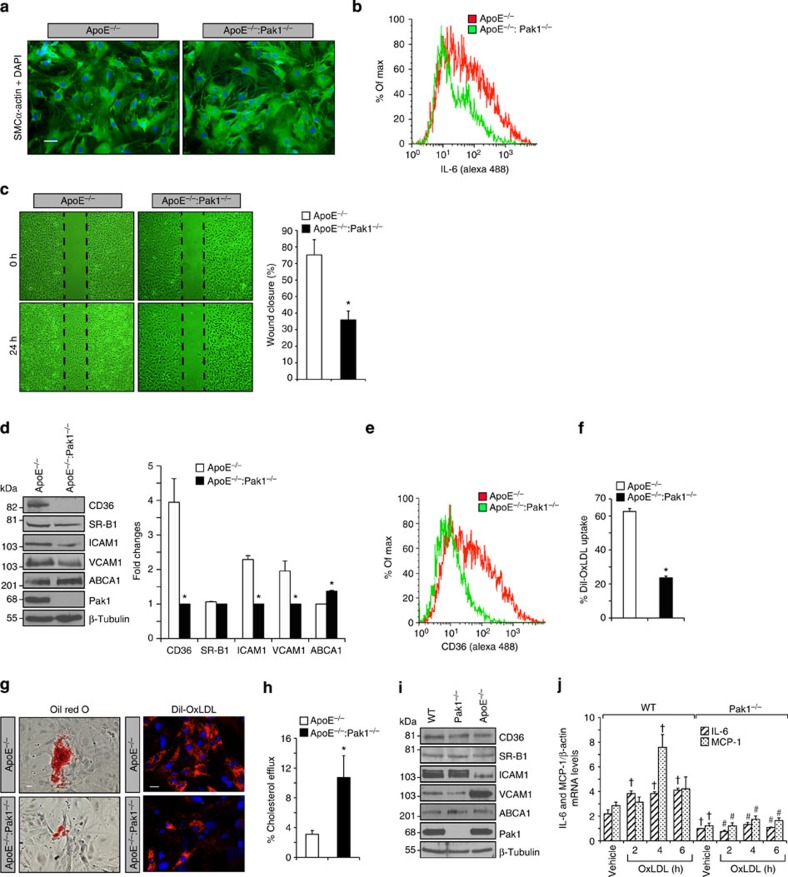
Pak1 regulates SMC migration and lesion formation. Aortic SMCs from ApoE^−/−^ and ApoE^−/−^:Pak1^−/−^ mice fed with WD for 16 weeks were isolated, cultured and tested for their purity using anti-SMCα-actin antibodies (**a**), analysed for IL-6 expression by FACS (**b**) and subjected to wound-healing migration assay (**c**). (**d**) Aortic SMC extracts from ApoE^−/−^ and ApoE^−/−^:Pak1^−/−^ mice were analysed by western blotting for the indicated proteins using their specific antibodies. FACS analysis of CD36 expression (**e**) and Dil-OxLDL uptake (**f**) are shown. SMCs isolated from ApoE^−/−^ and ApoE^−/−^:Pak1^−/−^ mice fed with WD for 16 weeks were either incubated with OxLDL or Dil-OxLDL for 6 h and stained with Oil red O (**g**, left panel) or observed under fluorescent microscope (**g**, right panel), or subjected to cholesterol efflux (**h**) as described in Methods. (**i**) Extracts of SMCs isolated from WT, Pak1^−/−^ and ApoE^−/−^ mice fed with CD were analysed by western blotting for the indicated proteins using their specific antibodies. (**j**) SMCs from WT and Pak1^−/−^ mice fed with CD were treated with OxLDL (10 μg ml^−1^) for the indicated time periods and RNA was isolated and analysed by qRT–PCR for IL-6 and MCP-1 levels. Bar graph in **c** represents quantification of three independent experiments. Bar graph in **d** represents the quantification of three western blottings each from a group of two pooled arteries. Data were presented as mean±s.d. and assessed by Student’s *t*-test. *P<0.01 versus ApoE^−/−^ mice; †*P*<0.01 versus WT vehicle control; #*P*<0.01 versus WT mice*+*OxLDL. Scale bars, 50 μm (**a**) and 100 and 50 μm (**g**, left and right panels, respectively).

Growth factor and cytokine production caused by dysfunctional endothelium may influence the phenotypic characteristics of SMCs resulting in their migration from media to intimal region where they can proliferate, synthesize and secrete extracellular matrix components, and thereby contribute to the progression of atherosclerotic lesions^5^. To understand the role of Pak1 in SMC migration and fibrofatty lesion formation, these cells were isolated from aortas of WD-fed ApoE^−/−^ and ApoE^−/−^:Pak1^−/−^ mice, their identity was confirmed by anti-SMCα-actin Ab staining (Fig. 5a) and examined for IL-6 expression by flow cytometry (Fig. 5b). A significant decrease in the number of SMCs positive for IL-6 was found in the aortas of ApoE^−/−^:Pak1^−/−^ mice as compared with that in ApoE^−/−^ mice. It was also observed that SMCs from ApoE^−/−^:Pak1^−/−^ mice exhibited decreased migration as compared with those from ApoE^−/−^ mice (Fig. 5c). Consistent with the findings in the aortic arch, SMCs from ApoE^−/−^:Pak1^−/−^ mice also showed decreased expression of CD36, SR-B1, ICAM1 and VCAM1 levels, and increased expression of ABCA1 levels, as compared with ApoE^−/−^ mice (Fig. 5d,e). To understand the potential contribution of SMCs in lesion progression, we examined their capacity of foam-cell formation by measuring Dil-OxLDL and/or OxLDL uptake and cholesterol efflux. We observed that SMCs from ApoE^−/−^:Pak1^−/−^ mice exhibited significantly decreased Dil-OxLDL and OxLDL uptake with a concomitant increase in cholesterol efflux as compared with SMCs from ApoE^−/−^ mice (Fig. 5f–h). To rule out the possibility that the observed changes in the levels of CD36, SR-B1, ICAM1, VCAM1 and ABCA1 in SMCs of ApoE^−/−^:Pak1^−/−^ mice were due to their derivation from smaller lesions, SMCs were isolated from CD-fed WT, Pak1^−/−^ and ApoE^−/−^ mice, and the above-listed molecules were measured. Although there were no major differences in the levels of these molecules in SMCs of WT and Pak1^−/−^ mice, SMCs from ApoE^−/−^ mice exhibited decreased ICAM1 with increased VCAM1 levels as compared with SMCs of WT or Pak1^−/−^ mice (Fig. 5i). To test the potential contribution of Pak1 in IL-6 and MCP-1 expression in SMC and their role in atherogenesis, SMCs from WT and Pak1^−/−^ mice were treated with and without OxLDL for the indicated time periods, and IL-6 and MCP-1 expression levels were measured. OxLDL induced both IL-6 and MCP-1 in SMCs of WT but not Pak1^−/−^ mice (Fig. 5j). These results further confirm the role of Pak1 in the induction of IL-6 and MCP-1, a potent cytokine and chemokine, respectively, in SMCs in response to atherosclerotic stimulant and their role in atherogenesis.

### Reduced trafficking of ApoE^−/−^:Pak1^−/−^ monocytes

**Figure 6:**
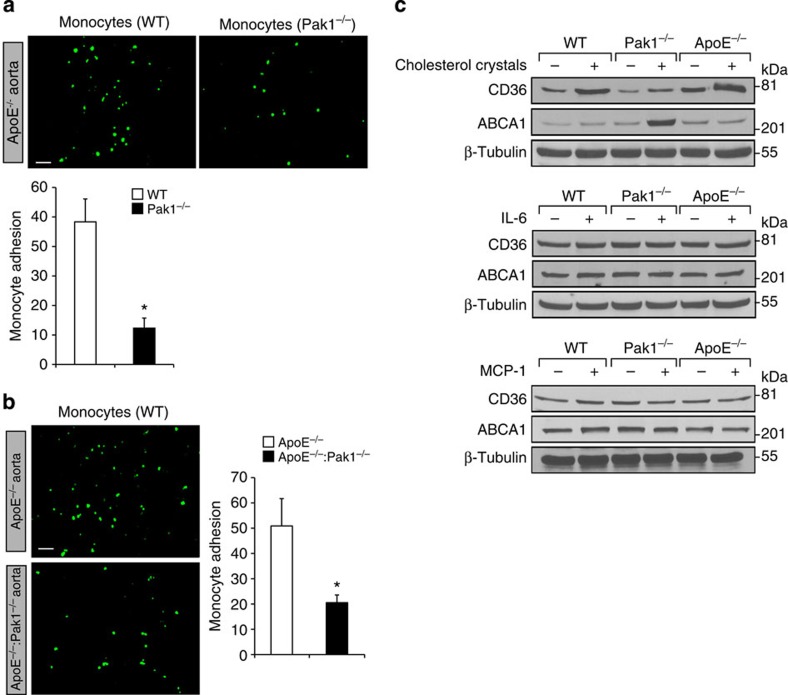
The lack of Pak1 diminishes monocyte adhesion to the aorta. (**a**) Aortas from 24-week-old ApoE^−/−^ mice were incubated with BCECF-labelled monocytes from C57BL/6 and Pak1^−/−^ mice, and the adherent monocytes were observed under a fluorescent microscope and counted. (**b**) Aortas from ApoE^−/−^ and ApoE^−/−^:Pak1^−/−^ mice fed with WD for 16 weeks were incubated with BCECF-labelled monocytes from C57BL/6 mice and the adherent cells were observed under a fluorescent microscope and counted. (**c**) Peritoneal macrophages from WT, ApoE^−/−^ and Pak1^−/−^ mice were treated with and without cholesterol (40 μg ml^−1^), IL-6 (20 ng ml^−1^) or MCP-1 (50 ng ml^−1^) for 6 h and cell extracts were prepared. Equal amounts of protein from control and each treatment were analysed by western blotting for CD36 and ABCA1 levels using their specific antibodies and normalized for β-tubulin. Bar graphs in **a** and **b** represent three independent experiments with three mice per group per experiment. Data were presented as mean±s.d. and assessed by Student’s *t*-test. *P<0.01 versus WT or ApoE^−/−^ mice. Scale bars, 20 μm (**a**,**b**).

**Figure 7:**
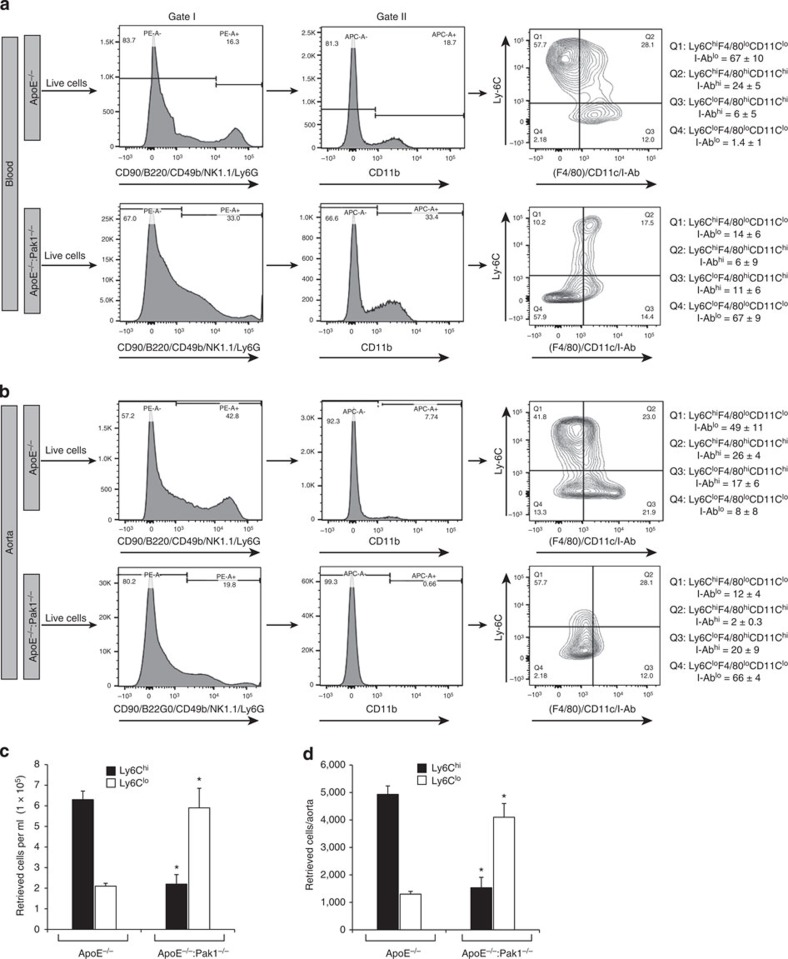
Monocyte subtypes in WD-fed ApoE^−/−^ and ApoE^−/−^:Pak1^−/−^ mice. (**a**) Blood was collected from ApoE^−/−^ and ApoE^−/−^:Pak1^−/−^ mice fed with WD, peripheral blood mononuclear cells were collected by density-gradient centrifugation, washed with RBC lysis buffer, resuspended into FACS buffer, blocked with mouse serum, washed with FACS buffer and incubated with anti-CD90-PE, anti-CD45R(B220)-PE, anti-CD49b-PE, anti-NK1.1-PE, anti-Ly6G-PE, anti-CD11b-APC, anti-Ly6C-FITC, anti-F4/80-Biotin, anti-I-Ab-Biotin and anti-CD11c-Biotin antibodies. After incubation with the required fluorochrome-conjugated streptavidin-PerCP-Cy5.5 antibodies and washings, the cells were resuspended into sorting buffer and subjected to FACS analysis. The gating strategy is indicated as follows. Live cells (selected based on higher forward scatter and lower side scatter) were gated as PE^−^ cells (in gate I). CD11b monocytes were gated as APC^+^ cells from PE^−^ cells (in gate II). CD11b^+^ monocytes were gated as Ly6C^hi^F4/80^lo^CD11c^lo^I-Ab^lo^, Ly6C^hi^F4/80^hi^CD11c^hi^I-Ab^hi^, Ly6C^lo^F4/80^hi^CD11c^hi^I-Ab^hi^ and Ly6C^lo^F4/80^lo^CD11c^lo^I-Ab^lo^. All gates were set using full-minus-one (FMO) controls. The mean percentages of each CD11b^+^ monocyte subpopulations for all mice are indicated in the respective gate. The average percentage of each CD11b^+^ monocyte subpopulations for all experiments are also listed. (**b**) Aortas from ApoE^−/−^ and ApoE^−/−^:Pak1^−/−^ mice fed with WD were collected, digested with a mixture of collagenase I, collagenase XI, Dnase I and hyaluronidase, washed with Hank’s balanced salt solution, resuspended in FACS buffer and subjected to FACS as described in **a**. The mean percentages of each CD11b^+^ monocyte subpopulations for all mice are indicated in the respective gate. (**c**,**d**) Bar graphs represent the number of retrieved Ly6C^hi^ and Ly6C^lo^ cells in the blood and the aorta of WD-fed ApoE^−/−^ versus ApoE^−/−^:Pak1^−/−^ mice. Data were presented as mean±s.d. and assessed by Student’s *t*-test. *P<0.01 versus ApoE^−/−^ mice (*n*=3 with 3 mice per group per experiment).

Reduced number of macrophages within the aorta of ApoE^−/−^:Pak1^−/−^ mice may suggest that Pak1 might be involved in the regulation of monocyte migration or adhesion to the inflamed aorta. Therefore, to test the role of Pak1 in the recruitment of monocytes to the aorta, we performed short-term homing assay *ex vivo*. We detected a significant decrease in the adhesion of Pak1^−/−^ monocytes to ApoE^−/−^ mice aorta as compared with WT monocytes (Fig. 6a). To further test our hypothesis that Pak1 via mediating the expression of adhesion molecules such as ICAM1 and VCAM1 promotes the recruitment of neutrophils and monocytes into the inflamed aorta, we performed *ex-vivo* monocyte adhesion assays using ApoE^−/−^ and ApoE^−/−^:Pak1^−/−^ aortas. A decrease in the adhesion of WT monocytes to the ApoE^−/−^:Pak1^−/−^ aorta was observed as compared with ApoE^−/−^ aorta (Fig. 6b), suggesting that Pak1 might promote atherogenesis by enhancing monocyte migration and its adhesion to the aortic wall. As macrophages from ApoE^−/−^:Pak1^−/−^ mice showed decreased uptake and increased efflux of OxLDL, we wanted to test whether Pak1 has any role in the modulation of scavenger receptor, CD36 and cholesterol reverse transporter ABCA1 levels. To address this assumption, we tested the effect of cholesterol esters, IL-6 and MCP-1 on CD36 and ABCA1 levels in the peritoneal macrophages of WT, ApoE^−/−^ and Pak1^−/−^ mice. It is exciting to note that although IL-6 and MCP-1 had no effect on the expression of these molecules, cholesterol esters increased CD36 expression in both WT and ApoE^−/−^ mice as compared with Pak1^−/−^ mice (Fig. 6c). Conversely, cholesterol esters enhanced the expression of ABCA1 in Pak1^−/−^ mice as compared with WT or ApoE^−/−^ mice. These findings indicate that Pak1 while negatively regulating the levels of ABCA1 enhances the expression of CD36 by cholesterol esters in macrophages. These results also infer that Pak1 by enhancing the uptake of modified lipids and decreasing their efflux plays an important role in foam-cell formation in response to WD. As IL-6 and MCP-1 levels were lower in macrophages of Pak1^−/−^ mice as compared with ApoE^−/−^ mice and these molecules are associated with macrophage M1 polarization, we determined the monocyte subtypes in the whole blood and aorta of WD-fed ApoE^−/−^ and ApoE^−/−^:Pak1^−/−^ mice. CD11b^+^ cells were identified in gate II as monocytes. The monocytes detected in gate II were divided into four phenotypically distinct subpopulations, that is, Ly6C^hi^F4/80^lo^CD11c^lo^I-Ab^lo^, Ly6C^hi^F4/80^hi^CD11c^hi^I-Ab^hi^, Ly6C^lo^F4/80^hi^CD11c^hi^I-Ab^hi^ and Ly6C^lo^F4/80^lo^CD11c^lo^I-Ab^lo^. We found that the number of Ly6C^hi^ monocytes were threefold lower in both the blood and aorta of ApoE^−/−^:Pak1^−/−^ mice as compared with that in ApoE^−/−^ mice (Fig. 7a–d). In contrast, the number of Ly6C^lo^ monocytes were substantially higher in the blood and aorta of ApoE^−/−^:Pak1^−/−^ mice as compared with that in ApoE^−/−^ mice (Fig. 7a–d). These findings reveal that Pak1 enhances proinflammation in response to WD feeding and influences atherogenesis, as Ly6C^hi^ monocytes are linked to disease progression and Ly6C^lo^ monocytes are associated with disease regression^27^,^28^.

**Figure 8:**
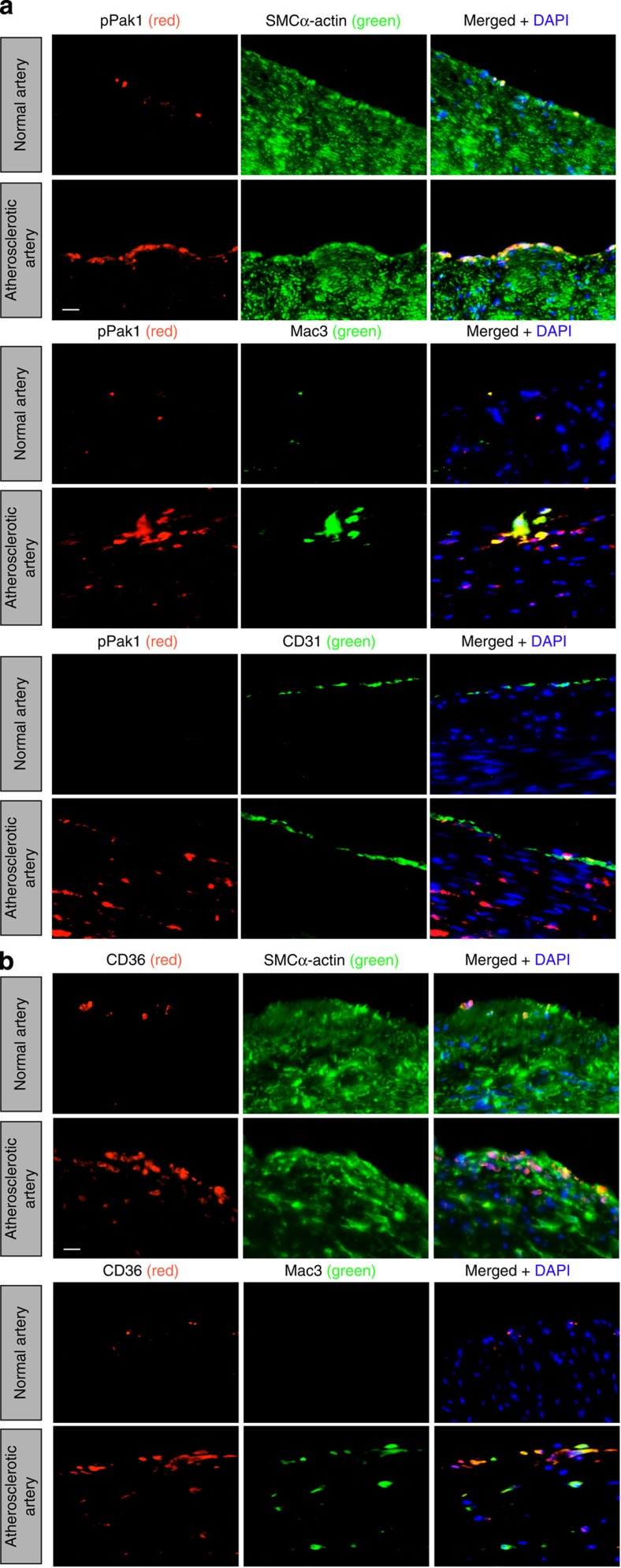
Human atherosclerotic arteries exhibit increased Pak1 phosporylation. (**a**,**b**) Paraffin-embedded human normal and atherosclerotic artery sections were stained for pPak1 (red) along with SMCα-actin (green), Mac3 (green) or CD31 (green), (**a**) or for CD36 (red) along with SMCα-actin or Mac3 (green) (**b**). Scale bars, 20 μm (**a**,**b**).

To determine whether Pak1 is playing any role in human atherosclerotic plaque development, we obtained normal and atherosclerotic human left-anterior descending artery sections and stained for pPak1 in combination with SMCα-actin, Mac3 or CD31. Consistent with our observations in ApoE^−/−^ mice, we found an increased Pak1 phosphorylation in SMCs and macrophages residing in the human atherosclerotic vessels as compared with that in non-atherosclerotic vessels (Fig. 8a). Very few endothelial cells were stained for pPak1 in human atherosclerotic lesions (Fig. 8a). As cholesterol esters induced CD36 expression in WT and ApoE^−/−^ but not in Pak1^−/−^ macrophages, we also stained the human normal and atherosclerotic artery sections for CD36 in combination with SMCα-actin or Mac3. Increased CD36 expression was observed in both SMCs and macrophages of atherosclerotic artery sections as compared with that in normal artery sections (Fig. 8b).

## Discussion

Pak1 plays a plethora of functions in various cellular processes, including chromatin remodelling^29^, actin cytoskeleton dynamics^30^, cell cycle progression^31^ and apoptosis^32^, and it does so by acting as a meeting point of many signalling pathways^6^,^7^,^8^,^9^,^10^,^11^,^12^,^13^. The primary initiating event in the pathogenesis of atherosclerosis is the accumulation of LDL in the subendothelial matrix because of damaged endothelium and some reports have shown that Pak activation stimulates paracellular pore formation leading to increased vascular permeability in response to cellular and atherogenic stimuli^15^,^16^. Furthermore, *in-vitro* studies have demonstrated that Pak1 mediates the downregulation of scavenger receptor B1 promoter activity, suggesting its role in cholesterol trafficking^14^. In light of these findings, we tested the hypothesis that Pak1 plays a role in atherogenesis using ApoE^−/−^ mice as an experimental model, as these mice develop the entire spectrum of lesions observed during atherogenesis and the lesions are similar to those in humans^18^.

Depletion of Pak1 resulted in reduced lesion formation in the aortic root and aorta of WD-fed ApoE^−/−^ mice. We observed a decrease in the plasma levels of IL-6 and MCP-1 in ApoE^−/−^:Pak1^−/−^ mice as compared with that in ApoE^−/−^ mice and these levels correlated with its decreased expression both in macrophages and SMCs of these mice. Experimental and epidemiological findings point out a prominent role for inflammation in the initiation and progression of atherogenesis^31^,^32^. IL-6 is an acute proinflammatory cytokine and it can be produced by endothelial cells, SMCs and activated macrophages^26^,^33^,^34^,^35^. It has been demonstrated that IL-6 induces the expression of MCP-1 in macrophages^35^. MCP-1 plays a vital role in the progression of atherosclerosis by increasing macrophage content and Ox-LDL accumulation^36^. Many studies have also demonstrated that IL-6 enhances the expression and secretion of various adhesion molecules and cytokines by vascular cells and cause the proliferation and migration of SMCs^37^,^38^. In the present study, we show that lack of Pak1 abrogates monocyte migration and their adhesion to aortas *ex vivo*. Based on all these observations, it can be inferred that Pak1 contributes to the pathogenesis of atherosclerosis by influencing many signalling events, including the expression of cytokines such as IL-6, chemokines such as MCP-1 and adhesion molecules such as VCAM1 and ICAM1, thereby recruiting monocytes to the aortic wall and the migration of SMCs from medial to intimal region. Pak1 plays an essential role in cell migration^39^. As increased migration and proliferation capacities are the characteristic features of dedifferentiated SMC phenotype^40^, the decreased migration of SMC from WD-fed ApoE^−/−^:Pak1^−/−^ mice as compared with SMC of ApoE^−/−^ mice may also infer that Pak1 plays a role in SMC phenotype switching during atherogenesis. Furthermore, Pak1 appears to be involved in enhancing inflammation via promoting macrophage M1 polarization, as the number of Ly6C^hi^ monocytes were found higher in both blood and aorta of ApoE^−/−^ mice as compared with that in ApoE^−/−^:Pak1^−/−^ mice. The role of Pak1 in macrophage M1 polarization can also be supported by the observation that its deficiency led to a significant increase in Ly6C^lo^ monocytes, which appear to be associated with inflammation clearance and disease regression^27^,^28^. In this regard, it should be pointed out that a recent study has demonstrated that Pak1 via mediating nuclear factor-κB activation plays a role in IL-6 and MCP-1 expression in macrophages and their M1 polarization^41^.

The role of CD36 on OxLDL uptake and foam-cell formation in atherogenesis has been well studied^19^,^20^,^21^. Accumulating body of evidence suggests that CD36 is a predominant scavenger receptor involved in the recognition and internalization of OxLDL and is instrumental in the formation of foam cells^20^,^42^. In this study, we have demonstrated that Pak1-deficient aortas as well as macrophages express reduced levels of CD36, leading to decreased capacity of both SMC and macrophages to take up OxLDL and form foam cells, and this may explain, at least partly, for a reduction in plaque burden in ApoE^−/−^:Pak1^−/−^ mice. The finding that cholesterol esters induce the expression of CD36 in the macrophages of WT and ApoE^−/−^ mice but not Pak1^−/−^ mice provides strong evidence that Pak1 mediates CD36 expression. This may imply that Pak1 might be involved in the activation of transcriptional factors such as PPARγ that has been shown to play a major role in CD36 expression^43^. In this aspect, previous studies from other laboratories have clearly demonstrated that p38MAPK, a downstream effector of Pak1, mediates the phosphorylation and activation of PPARγ coactivator, PGCα^44^,^45^. During atherogenesis, SMCs migrate from media to intima, where they can proliferate, synthesize and secrete extracellular matrix components, and provide a cushion for transition from fatty streak to more advanced fibro-fatty atheroma formation^5^,^46^. In this aspect, our findings also demonstrate that lack of Pak1 in ApoE^−/−^ mice show a significant decrease in CD36 expression and Dil-OxLDL/OxLDL uptake by SMCs, suggesting the role of Pak1 in SMC-specific foam-cell formation and their potential role in the progression of atherogenesis. In regard to the role of SR-B1 in atherogenesis, while some studies show that its levels correlate with cholesterol efflux^47^, other studies report an increase in its expression by atherogenic stimulants^48^. However, in the present study we found that WD feeding, although having no major effect on the levels of SR-B1 in macrophages, decreased its levels slightly in SMCs and aortic arches of ApoE^−/−^:Pak1^−/−^ mice compared with that in ApoE^−/−^ mice. Thus, these observations may suggest a differential role of SR-B1 in SMCs versus macrophages in OxLDL uptake and/or cholesterol efflux.

Efflux of cholesterol from peripheral tissues and its transport to liver by HDLs is a very well-established anti-atherogenic mechanism^49^. Several pathways of cholesterol removal from tissues, including aqueous diffusion, and ApoE- and ABCA1-mediated efflux play a role in the reverse cholesterol transport^25^,^50^,^51^,^52^. Our findings on ABCA1 expression in macrophages and SMCs show that Pak1 depletion increases ABCA1 levels in ApoE^−/−^ mice, thereby enhancing the cholesterol efflux from these cells, and it could be one of the mechanisms of decreased atherogenesis in ApoE^−/−^:Pak1^−/−^ mice. The observation that the lack of Pak1^−/−^ enhances ABCA1 and ABCG1 expression in macrophages reveals that Pak1 negatively regulates cholesterol efflux. The findings that both IL-6 and MCP-1 levels were found to be decreased in Pak1^−/−^ mice compared with WT mice that were maintained on CD may indicate that the reduction in the levels of scavenger receptors and adhesion molecules in both SMCs and macrophages of ApoE^−/−^:Pak1^−/−^ mice compared with ApoE^−/−^ mice in response to WD feeding were not due to consequences of lower plasma lipids or smaller plaques. Instead, these findings suggest that both IL-6 and MCP-1 may be involved in the regulation of these molecules, thereby causing lower SMC and macrophage migration, reduced lipid uptake and less foam-cell formation in ApoE^−/−^:Pak1^−/−^ mice. In addition, as Pak1 deficiency led to upregulation of reverse cholesterol transporters in ApoE^−/−^ mice in response to WD feeding, it may be suggested that Pak1 exerts a negative modulatory effect on these transporters and thereby may promote lipid retention in inflamed arteries causing atherogenesis.

In human atherosclerotic plaques, we observed an increased Pak1 phosphorylation along with CD36 expression in both macrophages and SMCs, whereas Pak1 phosphorylation and CD36 expression were undetectable in non-atherosclerotic vessels. These findings clearly imply that Pak1 is one of the critical protein kinases involved in the pathogenesis of human atherosclerosis. In summary, the present study demonstrates a role for Pak1 in the initiation and progression of atherogenesis, most probably through expression and release of IL-6 and MCP-1 by the inflamed artery, which then leads to increased trafficking of monocytes and SMCs to the intimal region resulting in enhanced Pak1-mediated CD36 expression causing increased uptake of OxLDL with decreased cholesterol efflux, which often culminates in heightened plaque burden in ApoE^−/−^ mice.

## Methods

### Reagents

Cholesterol (C8667), collagenase I (C9891), collagenase XI (C7657), DNase I (D5307), hyaluronidase (H3506), myelin basic protein (MBP; M1891) and anti-SMα-actin Abs (A2547) were purchased from Sigma-Aldrich (St. Louis, MO). Anti-CD36 (SC-260), anti-ICAM1 (SC-1511-R), anti-Mac3 (SC-19991), anti-SR-B1 (SC-67099), anti-β-tubulin (SC-9104) and anti-VCAM1 (SC-8304) Abs were obtained from Santa Cruz Biotechnology, Inc. (Santa Cruz, CA). Anti-ABCA1 (ab18180) and anti-pPak1 (ab2477) Abs were purchased from Abcam (Cambridge, MA). Anti-Pak1 (2602), anti-Pak2 (2608), anti-Pak3 (2609) and anti-Pak4 (3242) Abs were obtained from Cell Signaling Technology (Beverly, MA). Apolipoproteins (BT927), high TBAR Dil-OxLDL (BT-920) and high TBAR OxLDL (BT-910) were bought from Biomedical Technologies (Stoughton, MA). Anti-CD11b-APC (553312), anti-CD49b-PE (553858), anti-I-A(b)-biotin (550553), anti-IL-6 (559068), anti-Ly6C-FITC (553104), anti-Ly6G-PE (551461) and anti-NK-T-PE (550082) Abs were purchased from BD Pharmingen (San Jose, CA). Anti-CD11c-biotin (13-0114), anti-CD45R(B220)-PE (12-0452), anti-CD90.2-PE (12-0902) and anti-F4/80-biotin (13-4801) Abs were bought from eBiosciences (San Diego, CA). The ABC kit, DAB kit and Vectashield mounting medium were obtained from Vector Laboratories, Inc. (Burlingame, CA). Hoechst 33342 (3570) and Prolong Gold antifade mounting medium (P36930) were purchased from Invitrogen (Grand Island, NY). Recombinant mouse MCP-1 (470-JE) and mouse/rat MCP-1 quantikine enzyme-linked immunosorbent assay (ELISA) kit (MJE00) were bought from R&D Systems (Minneapolis, MN). [γ-^32^P]-ATP (S.A. 3,000 Ci mmol^−1^) was from MP Biomedicals (Irvine, CA). [^3^H]-Cholesterol (S.A. 53 Ci mmol^−1^) was obtained from Perkin Elmer (Waltham, MA). The enhanced chemiluminescence western blotting detection reagents (RPN2106), protein A-Sepharose (CL-4B) and protein G-Sepharose (CL-4B) beads were bought from GE Healthcare Lifesciences (Piscataway, NJ).

### Animals

ApoE^−/−^ mice (stock number 002052, Jackson Labs, Bar Harbor, ME) were crossed with Pak1^−/−^ mice (Mutant Mouse Regional Resource Center, UNC-Chapel Hill, Chapel Hill, NC) to obtain ApoE^−/−^:Pak1^−/−^ mice and both the strains were on C57BL/6 background. The F2 littermates were used in the study. Mice were bred and maintained according to the guidelines of the Institutional Animal Care and Use Facility of the University of Tennessee Health Science Center, Memphis, TN. Female and male ApoE^−/−^ mice and control C57BL/6 mice (Jackson Labs) were kept on a CD and were used at 24 weeks of age. Alternatively, male ApoE^−/−^ and ApoE^−/−^:Pak1^−/−^ mice were fed WD (21% fat and 0.2% cholesterol, Harlan Teklad, Harlan Laboratories, Indianapolis, IN) for 16 weeks starting at 8 weeks of age and used at 24 weeks of age. The Institutional Animal Care and Use Committee of the University of Tennessee Health Science Center, Memphis, TN, approved all the experiments involving animals.

### Human normal and atherosclerotic artery specimen

All human normal and atherosclerotic artery samples were collected following the protocols approved by the Institutional Review Boards^53^. These samples were considered to be deemed non-human subjects, as they were postmortem samples and therefore no informed consent was required. The postmortem samples were obtained from both the genders with age group ranging from 21 to 71 years.

### Isolation of SMCs

Aortic SMCs from C57BL/6, Pak1^−/−^, ApoE^−/−^ and ApoE^−/−^:Pak1^−/−^ mice were isolated by collagenase II/elastase digestion, grown in DMEM/F12 medium containing 10% fetal bovine serum, 100 units per ml penicillin and 100 μg ml^−1^ streptomycin in a humidified CO_2_ incubator at 37 °C and used between 3 and 7 passages. The purity of SMC was confirmed by anti-SMCα-actin staining.

### Isolation of peritoneal macrophages

To collect peritoneal macrophages, mice were injected intraperitoneally with 1 ml of autoclaved 4% thioglycolate. Four days later, the animals were anaesthetized with ketamine and xylazine, and the peritoneal lavage was collected in DMEM. Cells were incubated at 3 × 10^5^ cells per cm^2^ in DMEM containing penicillin (100 units per ml) and streptomycin (100 μg ml^−1^). After 3 h, floating cells (mostly red blood cells (RBCs)) were removed by washing with cold PBS and the adherent cells (macrophages) were used for migration and adhesion assays, or for the isolation of total cellular RNA or protein.

### Cell migration

Macrophage migration was measured using a modified Boyden chamber method^54^. Macrophages from WD-fed ApoE^−/−^ and ApoE^−/−^:Pak1^−/−^ mice were suspended in DMEM medium and plated on Matrigel-coated 8-μm cell culture inserts at 5 × 10^4^ cells per insert. MCP-1 was added to a final concentration of 50 ng ml^−1^ to the lower chamber and the cells were incubated for 8 h at 37 °C. The inserts were then lifted, non-migrated cells on the upper surface of the membrane were removed with a cotton swab and the membrane was then fixed in methanol and stained with DAPI (4′,6-diamidino-2-phenylindole). The DAPI-positive cells on the lower surface of the membrane were counted under an inverted microscope (Carl Zeiss AxioVision AX10) and the cell migration was expressed as the number of cells per field of view. SMC migration was measured by wound-healing assay. Briefly, SMCs were plated at 2 × 10^5^ cells per ml in each chamber of the ibidi culture inserts, grown to full confluency and growth arrested. Following a 24-h growth-arresting period, the inserts were removed using sterile tweezers and 2 ml of DMEM containing 5 mM hydroxyurea was added to the culture dish, and incubation continued for 48 h. The migrated cells were observed under Nikon Eclipse TS100 microscope with × 10/0.25 magnification and the images were captured with a Nikon Digital Sight DS-L1 camera.

### Cell adhesion assay

The adhesion of peritoneal macrophages to mouse pancreatic endothelial cell monolayer was measured by a fluorometric method^55^. Briefly, peritoneal macrophages were labelled with 10 μM BCECF-AM in serum-free DMEM for 30 min. The labelled cells were plated at 8 × 10^4^ cells per well onto a quiescent monolayer of pancreatic endothelial cells and incubated for 2 h, at which time the non-adherent cells were washed off with PBS. The adherent cells were then lysed in 0.2 ml of 0.1 M Tris-HCl containing 0.1% Triton X-100 and the fluorescence intensity was measured in a SpectraMax Gemini XS spectrofluorometer (Molecular Devices) with excitation at 485 nm and emission at 535 nm. Cell adhesion was expressed as relative fluorescence units.

### Western blot analysis

After appropriate treatments, cell or tissue extracts were prepared and resolved by electrophoresis on 0.1% (w/v) SDS and 8% or 10% (w/v) polyacrylamide gels. The proteins were transferred electrophoretically onto a nitrocellulose membrane. After being blocked in 5% (w/v) non-fat dry milk, the membrane was incubated with the appropriate primary Abs, followed by incubation with horseradish peroxidase-conjugated secondary Abs. The antigen–Ab complexes were detected with the enhanced chemiluminescence detection reagent kit (GE Healthcare). Anti-CD36 (SC-260), anti-ICAM1 (SC-1511-R), anti-Mac3 (SC-19,991), anti-SR-B1 (SC-67,099), anti-β-tubulin (SC-9,104) and anti-VCAM1 (SC-8,304) Abs were used at 1:500 dilution and anti-Pak1 (2,602), anti-Pak2 (2,608), anti-Pak3 (2,609) and anti-Pak4 (3,242) Abs were used at 1:1,000 dilution. Secondary Abs were used at 1: 5,000 dilution. Original images of the blots are provided in Supplementary Figs 1–9.

### Pak assay

Cell or tissue extracts were analysed for Pak1-4 activities according to the method of Wang *et al.*^54^. Equal amount of protein (300 μg) from cell and tissue extracts was first immunoprecipated with Pak1-4 Abs (1 μg of Ab per 200 μg of protein) for overnight at 4 °C, at which 80 μl of Protein A/G sepharose CL4B beads were added and incubation continued for additional 2 h at room temperature with gentle rocking. The immunocomplexes were collected by centrifugation and washed three to four times with lysis buffer, followed by one washing with kinase buffer (20 mM Hepes, pH 7.6, 20 mM MgCl_2_, 0.1 mM sodium orthovanadate, 2 mM dithiothreitol) and resuspended in 30 μl of the same buffer consisting of 5 μg MBP, 20 μM ATP and 10 μCi of [γ^32^P]-ATP, and incubated at 30 °C for 30 min. At the end of incubation, the reaction was terminated by adding 15 μl of 4 × Laemmeli sample buffer and boiling for 5 min. The reaction mix was then separated by 0.1% SDS–10% PAGE and the [^32^P]-labelled MBP was visualized by autoradiography.

### Quantitative reverse transcriptase–PCR

Total cellular RNA was isolated from the aorta and peritoneal macrophages of CD-fed C57BL/6, Pak1^−/−^ and ApoE^−/−^, as well as WD-fed ApoE^−/−^ and ApoE^−/−^:Pak1^−/−^ mice using a RNeasy Fibrous Tissue Mini kit (Qiagen, Valencia, CA) or a RiboPure kit (Ambion, Austin, TX) as per the manufacturer’s instructions. Reverse transcription was performed with a High Capacity complementary DNA reverse-transcription kit following the supplier’s protocol (Applied Biosystems, Foster City, CA). The cDNA was then used as a template for PCR amplification using TaqMan Gene Expression Assays for mouse IL-6 (Mm00446190_m1), mouse IL-12 (Mm00434165_m1), mouse TNFα (Mm00443260_g1), mouse MCP-1 (Mm00441242_m1), mouse Rantes (Mm01302427_m1) and mouse β-actin (Mm02619580_g1). The amplification was carried out on 7,300 Real-Time PCR Systems (Applied Biosystems) using the following conditions: 95 °C for 10 min followed by 40 cycles at 95 °C for 15 s with extension at 60 °C for 1 min for all genes. The PCR amplifications were examined using the 7,300 Real-Time PCR system-operated SDS version 1.4 program and Delta Rn analysis method (Applied Biosystems).

### Plasma cytokines and lipid profiles

Blood was collected into BD Vacutainer Plus plasma tubes (catalogue number 367960, BD Biosciences) by cardiac puncture and centrifuged at 1,300*g* for 10 min at 4 °C, to collect the plasma. The plasma cytokine levels were measured using their respective ELISA kits (MEM-004A, Multi-Analyte ELISArray kit, Qiagen). The total cholesterol, HDL, LDL and TGs levels in the plasma were measured using Roche Diagnostics COBAS MIRA analyser using the manufacturer’s kits.

### FACS analysis

To detect CD36-positive cells, peritoneal macrophages and aortic SMCs isolated from ApoE^−/−^ and ApoE^−/−^:Pak1^−/−^ mice were washed with cold FACS buffer (2% BSA and 0.1% sodium azide in PBS) and incubated with rabbit anti-human CD36 Ab or isotype control Ab for 30 min on ice, washed three times with FACS buffer and after the last wash the cells were resuspended in 100 μl FACS buffer containing Alexa Fluor 488-conjugated goat anti-rabbit secondary Abs and incubated for 30 min on ice. After incubation, cells were washed three times with FACS buffer and resuspended in 200 μl fixation buffer (1% paraformaldehyde in FACS buffer) and analysed using a FACS calibur flow cytometer (BD Biosciences, Oxford, UK). In the case of IL-6, cells were fixed in fixation buffer and permeabilized in 0.1% Triton X-100 before incubating with rat anti-mouse IL-6 Ab. After washings with FACS buffer, Alexa Fluor 488-conjugated goat anti-rat secondary Ab was added and the incubation was continued for 30 min on ice. Cells were then washed and analysed by a flow cytometer.

To determine the monocyte subtypes, blood was collected by cardiac puncture into EDTA-coated vacutainers. The mononuclear cells were purified by density-gradient centrifugation. To isolate macrophages from the aorta, the aortas were collected, placed into a mixture of collagenase I, collagenase XI, DNase I and hyaluronidase, incubated at 37 °C for 1 h and cells were collected as described by Galkina *et al.*^55^. The cell suspensions were centrifuged at 500*g* for 15 min at 4 °C, the RBCs were lysed with lysis buffer and the resulting single-cell suspensions were washed with PBS supplemented with 0.2% (w/v) BSA and 1% (w/v) FCS. For visualization of monocytes, the cells were incubated first with 3% mouse serum for 30 min on ice. After washing with FACS buffer, cells were labelled with a cocktail of monoclonal Abs against T cells (CD90.2-PE), B cells (CD45R(B220)-PE), natural killer cells (CD49b-PE and NK-T-PE), granulocytes (Ly6G-PE), monocytes (CD11b-APC) and monocyte subsets (Ly6C-FITC, F4/80-biotin, I-A(b)-biotin and CD11c-biotin) as described by Swirski *et al.*^27^. The latter three Abs that were included in the cocktail mixture to identify the monocyte subsets also serve to differentiate macrophages and dendritic cells from monocytes. Cells were washed three times with FACS buffer and then incubated with flurochrome-conjugated secondary Abs as required. Controls included cells that were incubated with or without the flurochrome-conjugated primary Abs followed by flurochrome-conjugated secondary Abs as needed. Flurophore-positive gates were set with full-minus-one controls. Gating strategy was set with live cells→T cells^−^, natural killer cells^−^, lymphocytes^−^, granulocytes^−^, B cells^−^→CD11b^+^→Ly6C^+/−^→CD11c^+/−^, F4/80^+/−^, I-Ab^+/−^. The cells were analysed using a BD LSRII flow cytometer. Each measurement contained 2 × 10^5^ cells. The cell sorting was performed on a FACS AriaII cell sorter (BD Biosciences, San Diego, CA). Monocyte percentages were calculated from the percentage of cells within the monocyte gate of the mononuclear cell fraction. Within this population, subsets were identified as either Ly6C^hi^ or Ly6C^lo^ cells. Data were analysed with DIVA v8.1 software (BD Biosciences, San Diego, CA).

### Enface staining

Aortas were excised and stained for atherosclerotic lesions using 0.5% Oil red O stain prepared in 60% isopropanol^56^. The images were photographed using Nikon D7100 camera and the per cent surface areas occupied by the lesions were measured using the National Institutes of Health ImageJ.

### Aortic roots

For immunohistochemistry and immunofluorescence staining, mice were perfused with 4% paraformaldehyde (PFA) and the hearts were collected. Sequential 10-μm aortic root sections were cut from the point of appearance of the aortic valve leaflets with a Leica CM3050 S cryostat machine (Leica Biosystems, Wetzlar, Germany).

### Immunohistochemistry

Aortic root sections were fixed in an equal volume of cold acetone and methanol for 10 min, permeabilized in 0.2% Triton X-100 for 10 min and blocked with 3% BSA for 30 min. The sections were then incubated with anti-Mac3 primary Ab (1:200) overnight at 4 °C, which was followed by incubation with biotin-conjugated goat anti-rat secondary Ab (1:500) for 1 h at room temperature. The sections were incubated with the ABC (avidin–biotin complex) reagent for 30 min, developed with DAB (3,3'-diaminobenzidine) reagent (Vector Laboratories) and counterstained with haematoxylin. The sections were observed under a Nikon Eclipse 50i microscope with × 4/0.10 or × 10/0.25 magnification and the images were captured with a Nikon Digital Sight DS-L1 camera.

### Oil Red O staining

After fixing with 4% PFA, the aortic root sections were washed once with 60% isopropanol and stained with Oil red O (0.5% in 60% isopropanol) for 15 min, followed by counterstaining with haematoxylin. The images were captured as described above under immunohistochemistry.

### Immunofluorescence staining

The aortic root sections were fixed with acetone/methanol (1:1) for 10 min, permeabilized in 0.2% Triton X-100 for 10 min, blocked with 5% goat serum in 3% BSA for 1 h and incubated with rat anti-mouse Mac3 and normal rabbit IgG, or rat anti-mouse Mac3 and rabbit anti-human pPak1, or rat anti-mouse Mac3 and rabbit anti-human CD36, or mouse anti-mouse SMCα-actin and normal rabbit IgG, or mouse anti-mouse SMCα-actin and rabbit anti-human pPak1, or mouse anti-mouse SMCα-actin and rabbit anti-human CD36 Abs (all at a 1:100 dilution), followed by incubation with Alexa Fluor 488-conjugated goat anti-rat and Alexa Fluor 568-conjugated goat anti-rabbit or Alexa Fluor 488-conjugated goat anti-mouse and Alexa Fluor 568-conjugated goat anti-rabbit secondary Abs, respectively (all at 1:500 dilution). The human normal and atherosclerotic artery sections were deparaffinized with xylene and then treated with antigen-unmasking solution for 30 min at 99 °C. The sections were then permeabilized with 0.5% Triton-X100 for 15 min and after blocking in normal goat serum the sections were probed with mouse anti-mouse SMCα-actin and rabbit anti-human pPak1, or mouse anti-mouse SMCα-actin and rabbit anti-human CD36, or mouse anti-human Mac3 and rabbit anti-human pPak1, or mouse anti-human Mac3 and rabbit anti-human CD36, or mouse anti-human CD31 and rabbit anti-human pPak1 Abs (all at a 1:100 dilution), followed by incubation with Alexa Fluor 488-conjugated goat anti-mouse and Alexa Fluor 568-conjugated goat anti-rabbit secondary Abs (all at 1:500 dilution), respectively. The sections were observed under a Zeiss Inverted Microscope (Zeiss AxioObserver Z1; magnification at × 40/NA (numerical aperture) 0.6) and the fluorescence images were captured with a Zeiss AxioCam MRm camera using the microscope operating software and Image Analysis Software AxioVision 4.7.2 (Carl Zeiss Imaging Solutions GmbH).

### Dil-OxLDL uptake assay

Peritoneal macrophages and aortic SMCs isolated from ApoE^−/−^ and ApoE^−/−^:Pak1^−/−^ mice were treated with Dil-labelled OxLDL (10 μg ml^−1^) for 6 h at 37 °C. After washing with PBS, OxLDL uptake was measured by FACS Calibur flow cytometer (BD Biosciences, Oxford, UK) with an acquired capacity of 10,000 cells. The data were analysed using CellQuest software and Dil-OxLDL uptake is presented as percentage of total cells.

### Cholesterol efflux assay

Peritoneal macrophages and SMCs were plated in 12-well dishes at a density of 6 × 10^5^ cells per well. Cells were incubated with [^3^H]-cholesterol (1 μCi ml^−1^) for 24 h followed by extensive washings with PBS. Cells were then equilibrated in serum-free DMEM containing 0.2% fatty acid free-BSA for 2 h. After equilibration, medium was replaced with fresh DMEM containing 0.2% fatty acid free-BSA and 10 μg ml^−1^ of Apolipoprotein A–I, and incubation was continued for 4 h at 37 °C. Either Apolipoprotein A–I or HDL can be used as an acceptor of cholesterol in the cholesterol efflux assay. An aliquot of the efflux medium (100 μl) was removed for radioactivity determination. Cells were then rinsed with PBS, dried and isopropanol was added for overnight extraction of cholesterol at room temperature. An aliquot of the extract (100 μl) was collected for radioactivity determination. Cholesterol efflux was expressed as % of total cellular radioactivity released into the medium.

### Foam cell-formation assay

Aortic SMCs isolated from WD-fed ApoE^−/−^ and ApoE^−/−^:Pak1^−/−^ mice were incubated with OxLDL (10 μg ml^−1^) for 6 h. The cells were then fixed with 4% PFA for 30 min, stained with Oil red O stain for 10 min and counterstained with haematoxylin. Cell staining was observed under a Nikon Eclipse 50i microscope with × 4 magnification and images were captured with a Nikon Digital Slight DS-L1 camera

### *Ex-vivo* monocyte adhesion assay

The monocyte adhesion assay was performed according to the method of Smith *et al.*^57^. Briefly, aortas from 16-week WD-fed ApoE^−/−^ and ApoE^−/−^:Pak1^−/−^ mice were dissected out, opened longitudinally and pinned to a sterile agarose gel in serum-free DMEM. In parallel, CD115-positive monocytes were isolated using affinity beads (Miltenyi Biotec) from C57BL/6 or Pak1^−/−^ mice and labelled with 10 μM BCECF-AM. BCECF-labelled monocytes were coincubated with pinned aortas in DMEM for 1 h. Non-adhered monocytes were washed off with PBS and the number of monocytes adhered to the aorta were counted using a fluorescent microscope (Zeiss AxioObserver Z1; Magnification at × 40/NA 0.6), and the fluorescence images were captured with a Zeiss AxioCam MRm camera using the microscope operating software and Image Analysis Software AxioVision 4.7.2 (Carl Zeiss Imaging Solutions GmbH).

### Preparation of cholesterol crystals

Cholesterol crystals were prepared according to the method of Flynn *et al.*^58^. Briefly, cholesterol was dissolved in 95% ethanol (12.5 g l^−1^), heated at 60 °C for 2 h, filtered through Whatman filter paper and left at room temperature for crystallization. Crystals were collected by filtering, grinded with autoclaved mortar and pestle to yield 1–10 μm in size, and stored at −20 °C.

### Statistics

All the experiments were repeated three times with similar results. Data are presented as the mean±s.d. The treatment effects were analysed by Student’s *t*-test and *P*-values <0.05 were considered to be statistically significant. In the case of western blotting, immunohistochemistry, immunofluorescence and immunocomplex kinase assay, one set of the representative data is presented.

## Additional information

**How to cite this article:** Singh, N. K. *et al.* Disruption of *p21-activated kinase 1* gene diminishes atherosclerosis in apolipoprotein E-deficient mice. *Nat. Commun.* 6:7450 doi: 10.1038/ncomms8450 (2015).

## Supplementary Material

Supplementary Information Supplementary Figures 1-9

## Figures and Tables

**Table 1:** Effect of western diet on plasma lipids.

**Genotype**	**Diet**	**Weight (g)**	**Cholesterol (mg** **dl**^**−1**^)	**HDL (mg** **dl**^**−1**^)	**LDL (mg** **dl**^**−1**^)	**TGs (mg** **dl**^**−1**^)	***n***
ApoE^−/−^	WD	34±3	1209±77	687±55	480±114	206±62	8
ApoE^−/^:Pak1^−/−^	WD	32±7	1007±133*	578±193	222±161*	106±53	7

HDL, high-density lipoprotein; LDL, low-density lipoprotein; TG, triglyceride; WD, Western diet.

*P< 0.01 versus ApoE^−/−^ mice.

**Table 2:** **Effect of chow diet on plasma lipids.**

**Genotype**	**Diet**	**Weight (g)**	**Cholesterol (mg** **dl**^**−1**^)	**HDL (mg** **dl**^**−1**^)	**LDL (mg** **dl**^**−1**^)	**TGs (mg** **dl**^**−1**^)	***n***
WT	CD	24±2	91±5	80±7	1.13±0.8	74±20	9
Pak1^−/−^	CD	26±2	90±2	78±13	1.25±0.7	77±21	9
ApoE^−/−^	CD	25±1	238±46^†^	161±55^†^	51.3±18^†^	133±45^†^	9
ApoE^−/^:Pak1^−/−^	CD	23±2	217±38^†^	133±30^†^	62.8±19^†^	110±33^†^	7

CD, chow diet; HDL, high-density lipoprotein; LDL, low-density lipoprotein; TG, triglyceride; WT, wild type.

†*P*< 0.01 versus WT or Pak1^−/−^ mice.

## References

[b1] Libby P., Ridker P. M. & Hansson G. K. Progress and challenges in translating the biology of atherosclerosis. Nature 473, 317–325 (2011). 21593864 10.1038/nature10146

[b2] Ross R. Atherosclerosis—an inflammatory disease. N. Engl. J. Med. 340, 115–126 (1999). 9887164 10.1056/NEJM199901143400207

[b3] Steinberg D. A critical look at the evidence for the oxidation of LDL in atherogenesis. Atherosclerosis 131, S5–S7 (1997). 9253467 10.1016/s0021-9150(97)06115-7

[b4] Schwartz S. M. Smooth muscle migration in atherosclerosis and restenosis. J. Clin. Invest. 100, S87–S89 (1997). 9413408

[b5] Bennett M. R. Apoptosis of vascular smooth muscle cells in vascular remodelling and atherosclerotic plaque rupture. Cardiovasc. Res. 41, 361–368 (1999). 10341835 10.1016/s0008-6363(98)00212-0

[b6] Bagrodia S. & Cerione R. A. Pak to the future. Trends Cell Biol. 9, 350–355 (1999). 10461188 10.1016/s0962-8924(99)01618-9

[b7] Hofmann C., Shepelev M. & Chernoff J. The genetics of Pak. J. Cell. Sci. 117, 4343–4354 (2004). 15331659 10.1242/jcs.01392

[b8] Nikolic M. The pak1 kinase: an important regulator of neuronal morphology and function in the developing forebrain. Mol. Neurobiol. 37, 187–202 (2008). 18649038 10.1007/s12035-008-8032-1

[b9] Bokoch G. M. Biology of the p21-activated kinases. Annu. Rev. Biochem. 72, 743–781 (2003). 12676796 10.1146/annurev.biochem.72.121801.161742

[b10] Ke Y., Wang L., Pyle W. G., de Tombe P. P. & Solaro R. J. Intracellular localization and functional effects of P21-activated kinase-1 (Pak1) in cardiac myocytes. Circ. Res. 94, 194–200 (2004). 14670848 10.1161/01.RES.0000111522.02730.56

[b11] Liu J. *et al.* A betaPix Pak2a signaling pathway regulates cerebral vascular stability in zebrafish. Proc. Natl Acad. Sci. USA 104, 13990–13995 (2007). 17573532 10.1073/pnas.0700825104 PMC1955796

[b12] Nekrasova T. & Minden A. Role for p21-activated kinase PAK4 in development of the mammalian heart. Transgenic Res. 21, 797–811 (2012). 22173944 10.1007/s11248-011-9578-7

[b13] Radu M., Semenova G., Kosoff R. & Chernoff J. Pak signalling during the development and progression of cancer. Nat. Rev. Cancer 14, 13–25 (2014). 24505617 10.1038/nrc3645 PMC4115244

[b14] Hullinger T. G., Panek R. L., Xu X. & Karathanasis S. K. p21-activated kinase-1 (PAK1) inhibition of the human scavenger receptor class B, type I promoter in macrophages is independent of PAK1 kinase activity, but requires the GTPase-binding domain. J. Biol. Chem. 276, 46807–46814 (2001). 11585816 10.1074/jbc.M103176200

[b15] Orr A. W. *et al.* Matrix-specific p21-activated kinase activation regulates vascular permeability in atherogenesis. J. Cell Biol. 176, 719–727 (2007). 17312022 10.1083/jcb.200609008 PMC2064028

[b16] Stockton R. A., Schaefer E. & Schwartz M. A. p21-activated kinase regulates endothelial permeability through modulation of contractility. J. Biol. Chem. 279, 46621–46630 (2004). 15333633 10.1074/jbc.M408877200

[b17] Jhaveri K. A., Debnath P., Chernoff J., Sanders J. & Schwartz M. A. The role of p21-activated kinase in the initiation of atherosclerosis. BMC Cardiovasc. Disord. 12, 55 (2012). 22824149 10.1186/1471-2261-12-55 PMC3489605

[b18] Nakashima Y., Plump A. S., Raines E. W., Breslow J. L. & Ross R. ApoE-deficient mice develop lesions of all phases of atherosclerosis throughout the arterial tree. Arterioscler. Thromb. 14, 133–140 (1994). 8274468 10.1161/01.atv.14.1.133

[b19] Endemann G. *et al.* CD36 is a receptor for oxidized low density lipoprotein. J. Biol. Chem. 268, 11811–11816 (1993). 7685021

[b20] Febbraio M. *et al.* Targeted disruption of the class B scavenger receptor CD36 protects against atherosclerotic lesion development in mice. J. Clin. Invest. 105, 1049–1056 (2000). 10772649 10.1172/JCI9259 PMC300837

[b21] Kunjathoor V. V. *et al.* Scavenger receptors class A-I/II and CD36 are the principal receptors responsible for the uptake of modified low density lipoprotein leading to lipid loading in macrophages. J. Biol. Chem. 277, 49982–49988 (2002). 12376530 10.1074/jbc.M209649200

[b22] Huber S. A., Sakkinen P., Conze D., Hardin N. & Tracy R. Interleukin-6 exacerbates early atherosclerosis in mice. Arterioscler. Thromb. Vasc. Biol. 19, 2364–2367 (1999). 10521365 10.1161/01.atv.19.10.2364

[b23] Boring L., Gosling J., Cleary M. & Charo I. F. Decreased lesion formation in CCR2^−/−^ mice reveals a role for chemokines in the initiation of atherosclerosis. Nature 394, 894–897 (1998). 9732872 10.1038/29788

[b24] Amar J. *et al.* Interleukin 6 is associated with subclinical atherosclerosis: a link with soluble intercellular adhesion molecule 1. J. Hypertens. 24, 1083–1088 (2006). 16685208 10.1097/01.hjh.0000226198.44181.0c

[b25] Yvan-Charvet L. *et al.* Combined deficiency of ABCA1 and ABCG1 promotes foam cell accumulation and accelerates atherosclerosis in mice. J. Clin. Invest. 117, 3900–3908 (2007). 17992262 10.1172/JCI33372 PMC2066200

[b26] Tieu B. C. *et al.* An adventitial IL-6/MCP1 amplification loop accelerates macrophage-mediated vascular inflammation leading to aortic dissection in mice. J. Clin. Invest. 119, 3637–3651 (2009). 19920349 10.1172/JCI38308 PMC2786788

[b27] Swirski F. K. *et al.* Ly-6Chi monocytes dominate hypercholesterolemia-associated monocytosis and give rise to macrophages in atheromata. J. Clin. Invest. 117, 195–205 (2007). 17200719 10.1172/JCI29950 PMC1716211

[b28] Babaev V. R. *et al.* Macrophage deficiency of Akt2 reduces atherosclerosis in Ldlr null mice. J. Lipid Res. 55, 2296–2308 (2014). 25240046 10.1194/jlr.M050633 PMC4617132

[b29] Li F. *et al.* p21-activated kinase 1 interacts with and phosphorylates histone H3 in breast cancer cells. EMBO Rep. 3, 767–773 (2002). 12151336 10.1093/embo-reports/kvf157 PMC1084211

[b30] Edwards D. C., Sanders L. C., Bokoch G. M. & Gill G. N. Activation of LIM-kinase by Pak1 couples Rac/Cdc42 GTPase signalling to actin cytoskeletal dynamics. Nat. Cell Biol. 1, 253–259 (1999). 10559936 10.1038/12963

[b31] Zhao Z. S., Lim J. P., Ng Y. W., Lim L. & Manser E. The GIT-associated kinase PAK targets to the centrosome and regulates Aurora-A. Mol. Cell 20, 237–249 (2005). 16246726 10.1016/j.molcel.2005.08.035

[b32] Vadlamudi R. K. *et al.* Regulatable expression of p21-activated kinase-1 promotes anchorage-independent growth and abnormal organization of mitotic spindles in human epithelial breast cancer cells. J. Biol. Chem. 275, 36238–36244 (2000). 10945974 10.1074/jbc.M002138200

[b33] Hansson G. K. Inflammation, atherosclerosis, and coronary artery disease. N. Engl. J. Med. 352, 1685–1695 (2005). 15843671 10.1056/NEJMra043430

[b34] Paoletti R., Gotto A. M. Jr. & Hajjar D. P. Inflammation in atherosclerosis and implications for therapy. Circulation 109, III20–III26 (2004). 15198962 10.1161/01.CIR.0000131514.71167.2e

[b35] Biswas P. *et al.* Interleukin-6 induces monocyte chemotactic protein-1 in peripheral blood mononuclear cells and in the U937 cell line. Blood 91, 258–265 (1998). 9414293

[b36] Aiello R. J. *et al.* Monocyte chemoattractant protein-1 accelerates atherosclerosis in apolipoprotein E-deficient mice. Arterioscler. Thromb. Vasc. Biol. 19, 1518–1525 (1999). 10364084 10.1161/01.atv.19.6.1518

[b37] Romano M. *et al.* Role of IL-6 and its soluble receptor in induction of chemokines and leukocyte recruitment. Immunity 6, 315–325 (1997). 9075932 10.1016/s1074-7613(00)80334-9

[b38] Chava K. R. *et al.* CREB-mediated IL-6 expression is required for 15(S)-hydroxyeicosatetraenoic acid-induced vascular smooth muscle cell migration. Arterioscler. Thromb. Vasc. Biol. 29, 809–815 (2009). 19342597 10.1161/ATVBAHA.109.185777 PMC2724759

[b39] Sells M. A. *et al.* Human p21-activated kinase (Pak1) regulates actin organization in mammalian cells. Curr. Biol. 7, 202–210 (1997). 10.1016/s0960-9822(97)70091-5 9395435

[b40] Gomez D. & Owens G. K. Smooth muscle cell phenotypic switching in atherosclerosis. Cardiovasc. Res. 95, 156–164 (2012). 22406749 10.1093/cvr/cvs115 PMC3388816

[b41] Zhang W. *et al.* Polycomb-mediated loss of microRNA let-7c determines inflammatory macrophage polarization via PAK1-dependent NF-κB pathway. Cell Death Differ. 142, 1–11 (2014). 10.1038/cdd.2014.142 PMC4291490 25215948

[b42] Ross R. The pathogenesis of atherosclerosis: a perspective for the 1990s. Nature 362, 801–809 (1993). 8479518 10.1038/362801a0

[b43] Huang J. T. *et al.* Interleukin-4-dependent production of PPAR-gamma ligands in macrophages by 12/15-lipoxygenase. Nature 400, 378–382 (1999). 10432118 10.1038/22572

[b44] Puigserver P. *et al.* Cytokine stimulation of energy expenditure through p38 MAP kinase activation of PPARgamma coactivator-1. Mol. Cell 8, 971–982 (2001). 11741533 10.1016/s1097-2765(01)00390-2

[b45] Chan P. M., Lim L. & Manser E. PAK is regulated by PI3K, PIX, CDC42, and PP2Calpha and mediates focal adhesion turnover in the hyperosmotic stress-induced p38 pathway. J. Biol. Chem. 283, 24949–24961 (2008). 18586681 10.1074/jbc.M801728200 PMC3259815

[b46] Lusis A. J. Atherosclerosis. Nature 407, 233–241 (2000). 11001066 10.1038/35025203 PMC2826222

[b47] Maitra U. & Li L. Molecular mechanisms responsible for the reduced expression of cholesterol transporters from macrophages by low-dose endotoxin. Arterioscler. Thromb. Vasc. Biol. 33, 24–33 (2013). 23117655 10.1161/ATVBAHA.112.300049 PMC3545450

[b48] Kushiyama A. *et al.* Xanthine oxidoreductase is involved in macrophage foam cell formation and atherosclerosis development. Arterioscler. Thromb. Vasc. Biol. 32, 291–298 (2012). 22095983 10.1161/ATVBAHA.111.234559

[b49] Glomset J. A. The plasma lecithins:cholesterol acyltransferase reaction. J. Lipid. Res. 9, 155–167 (1968). 4868699

[b50] Westerterp M. *et al.* Atp-binding cassette transporters, atherosclerosis, and inflammation. Circ. Res. 114, 157–170 (2014). 24385509 10.1161/CIRCRESAHA.114.300738

[b51] Langer C. *et al.* Endogenous apolipoprotein E modulates cholesterol efflux and cholesteryl ester hydrolysis mediated by high-density lipoprotein-3 and lipid-free apolipoproteins in mouse peritoneal macrophages. J. Mol. Med. (Berl) 78, 217–227 (2000). 10933584 10.1007/s001090000096

[b52] Ji Y. *et al.* Scavenger receptor BI promotes high density lipoprotein-mediated cellular cholesterol efflux. J. Biol. Chem. 272, 20982–20985 (1997). 9261096 10.1074/jbc.272.34.20982

[b53] Funk S. D. *et al.* EphA2 activation promotes the endothelial cell inflammatory response: a potential role in atherosclerosis. Arterioscler. Thromb. Vasc. Biol. 32, 686–695 (2012). 22247258 10.1161/ATVBAHA.111.242792 PMC3325141

[b54] Wang D. *et al.* A role for Gab1/SHP2 in thrombin activation of PAK1: gene transfer of kinase-dead PAK1 inhibits injury-induced restenosis. Circ. Res. 104, 1066–1075 (2009). 19359598 10.1161/CIRCRESAHA.109.196691 PMC2814372

[b55] Galkina E. *et al.* Lymphocyte recruitment into the aortic wall before and during development of atherosclerosis is partially L-selectin dependent. J. Exp. Med. 203, 1273–1282 (2006). 16682495 10.1084/jem.20052205 PMC2121208

[b56] Nunnari J. J., Zand T., Joris I. & Majno G. Quantitation of oil red O staining of the aorta in hypercholesterolemic rats. Exp. Mol. Pathol. 51, 1–8 (1989). 2767215 10.1016/0014-4800(89)90002-6

[b57] Smith E. *et al.* Blockade of interleukin-17A results in reduced atherosclerosis in apolipoprotein E-deficient mice. Circulation 121, 1746–1755 (2010). 20368519 10.1161/CIRCULATIONAHA.109.924886 PMC2929562

[b58] Flynn G. L. *et al.* Cholesterol solubility in organic solvents. J. Pharm. Sci. 68, 1090–1097 (1979). 501527 10.1002/jps.2600680908

