# Supplementary material for: Disruption of p21-activated kinase 1 gene diminishes atherosclerosis in apolipoprotein E-deficient mice
Source: Nat Commun. 2015 Jun 24;6:7450. doi: 10.1038/ncomms8450 (PMC4480433; doi:10.1038/ncomms8450)
Supplement: Supplementary Information — Supplementary Figures 1-9 [file ncomms8450-s1.pdf]

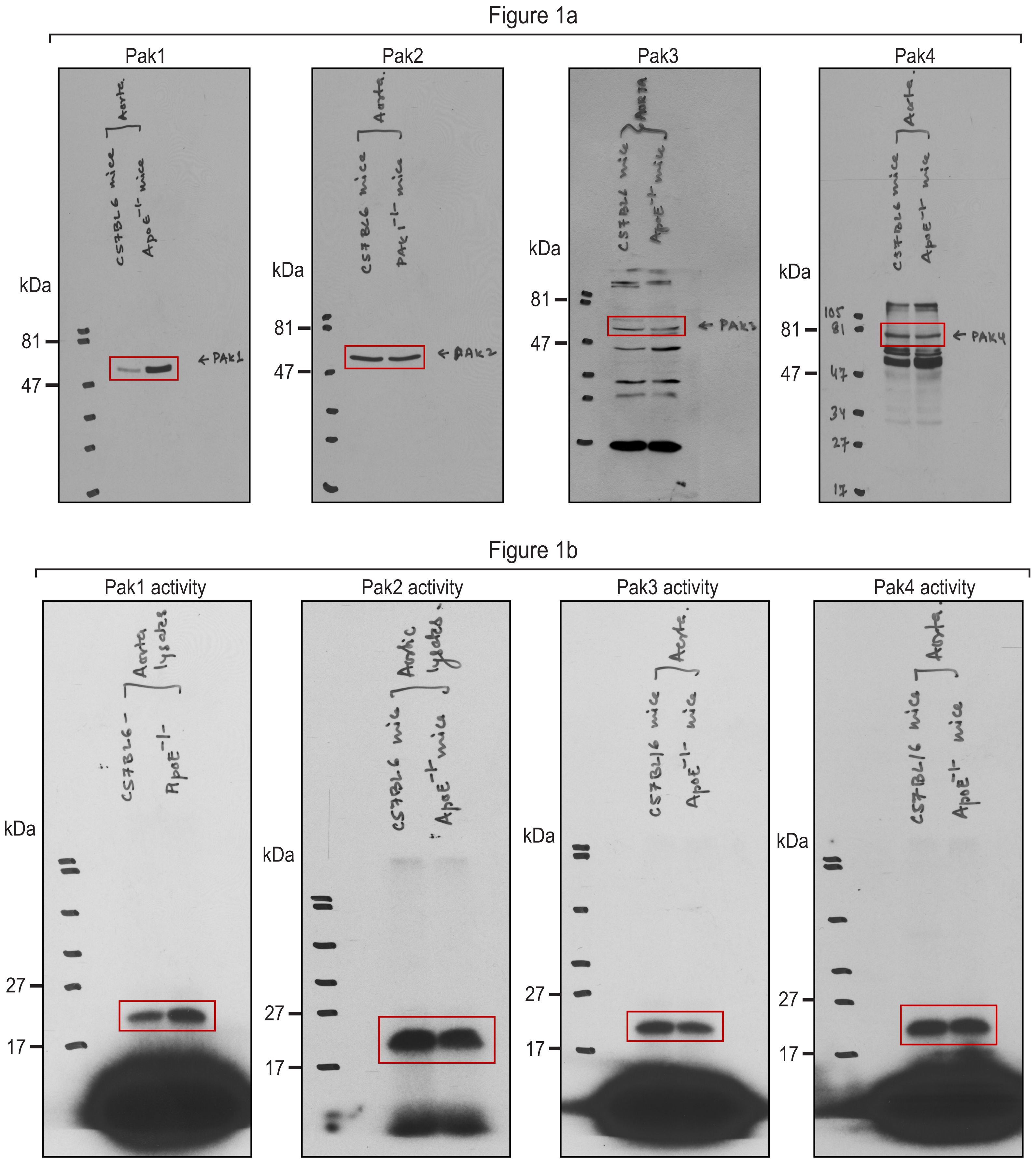

Supplementary Figure 1: Scans of immunoblots presented in Figure 1a & b.

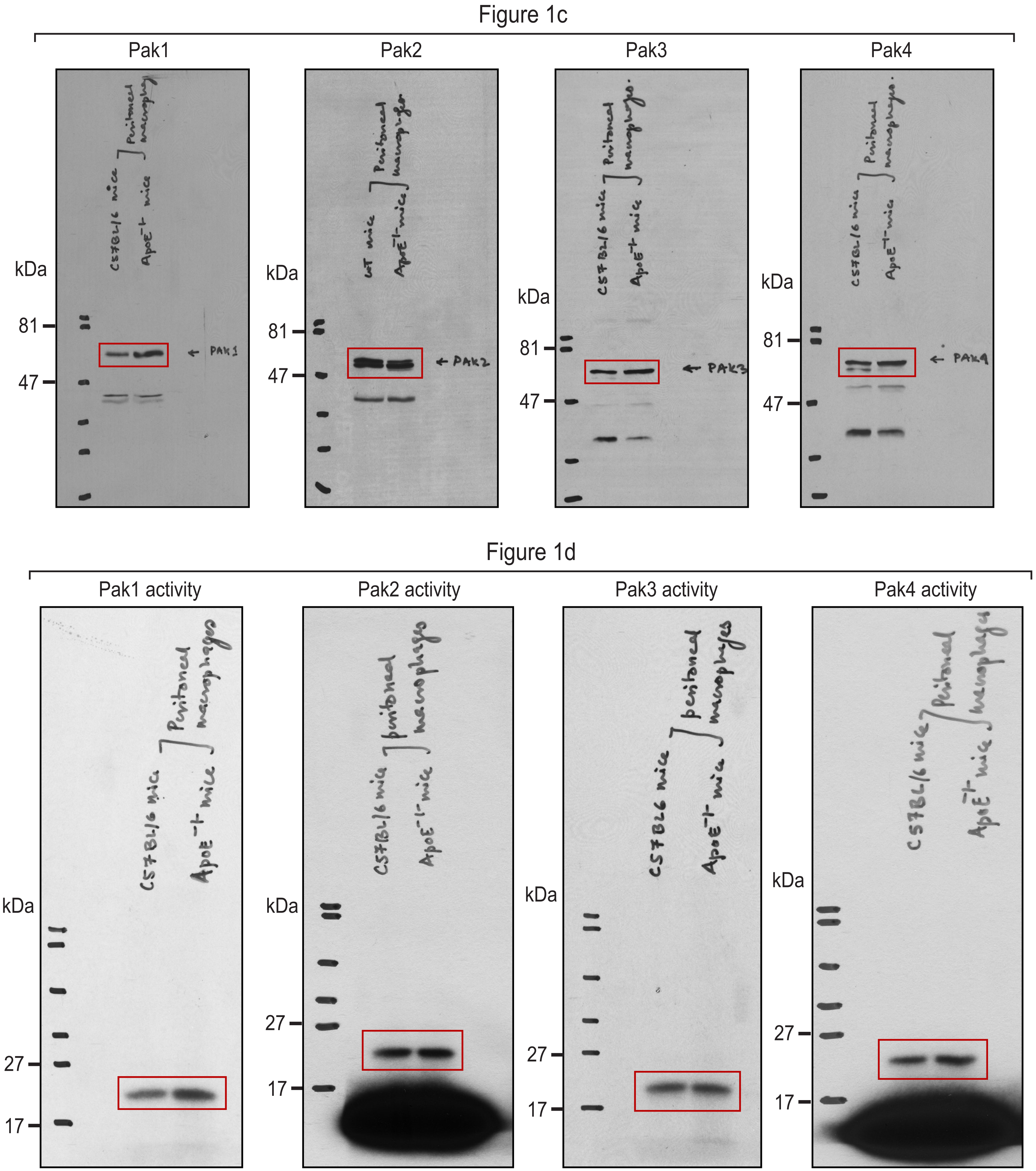

Supplementary Figure 2: Scans of immunoblots presented in Figure 1c & d.

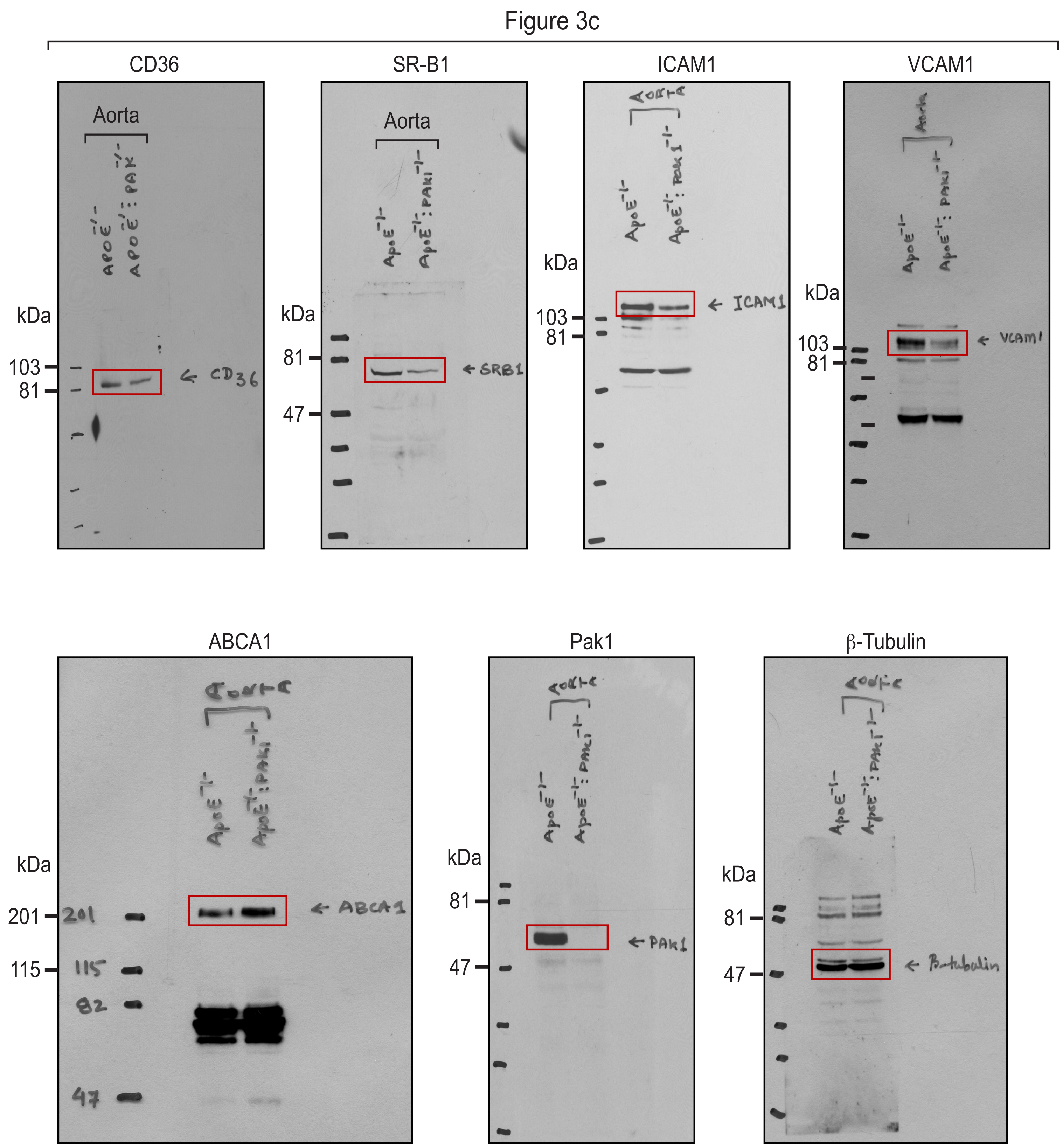

Supplementary Figure 3: Scans of immunoblots presented in Figure 3c.

Figure 3f

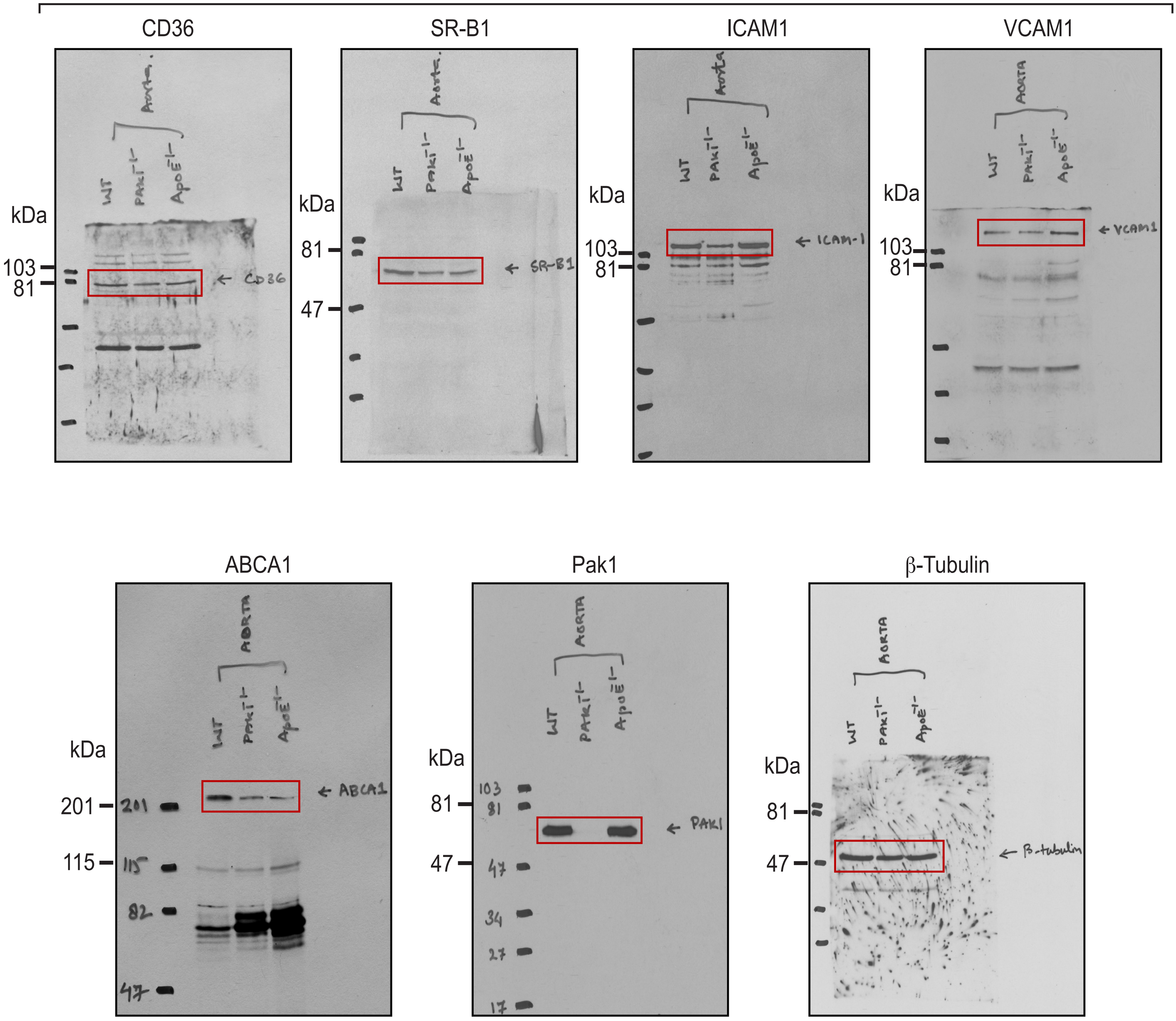

Supplementary Figure 4: Scans of immunoblots presented in Figure 3f.

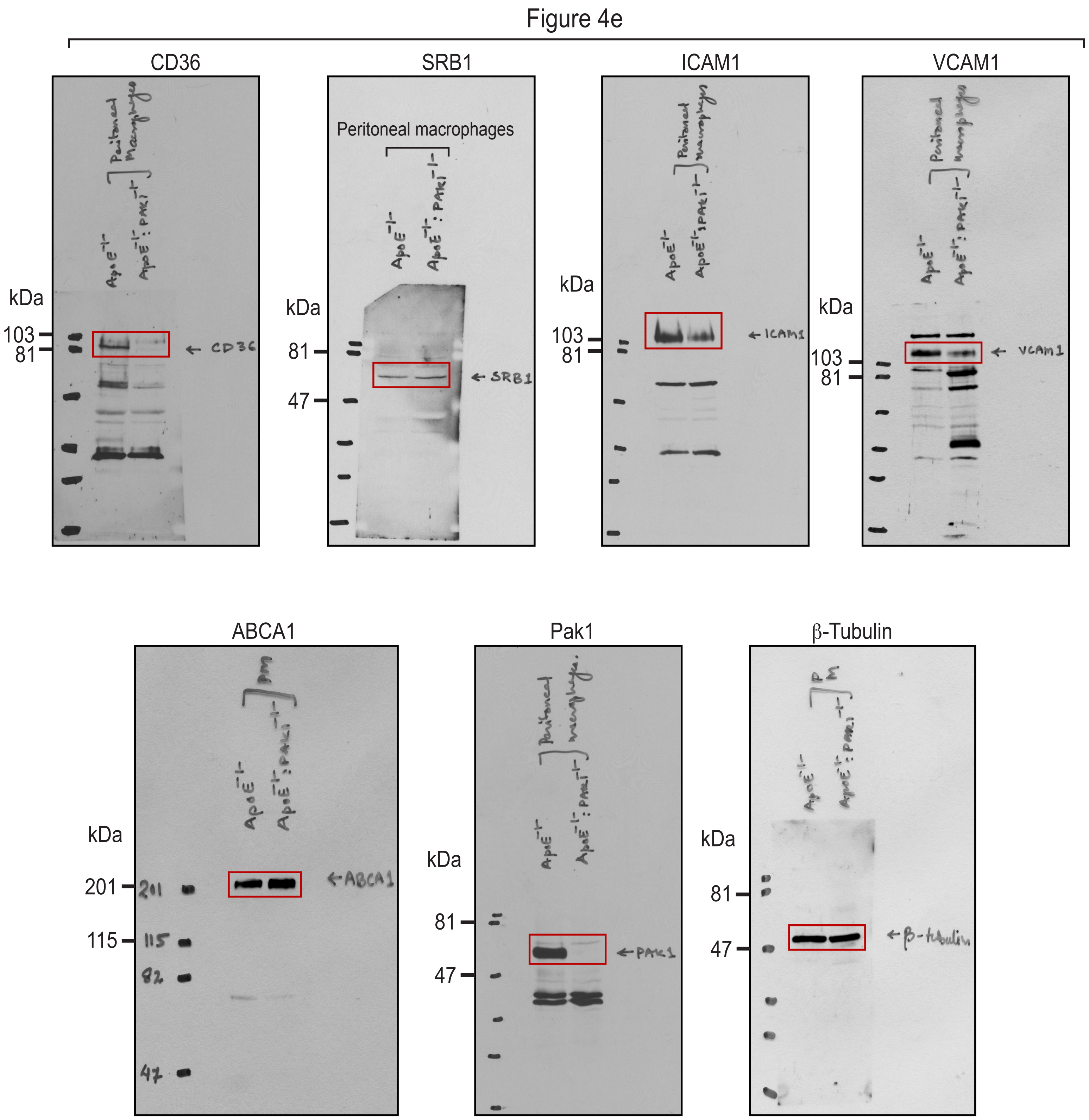

Supplementary Figure 5: Scans of immunoblots presented in Figure 4e.

Figure 4k

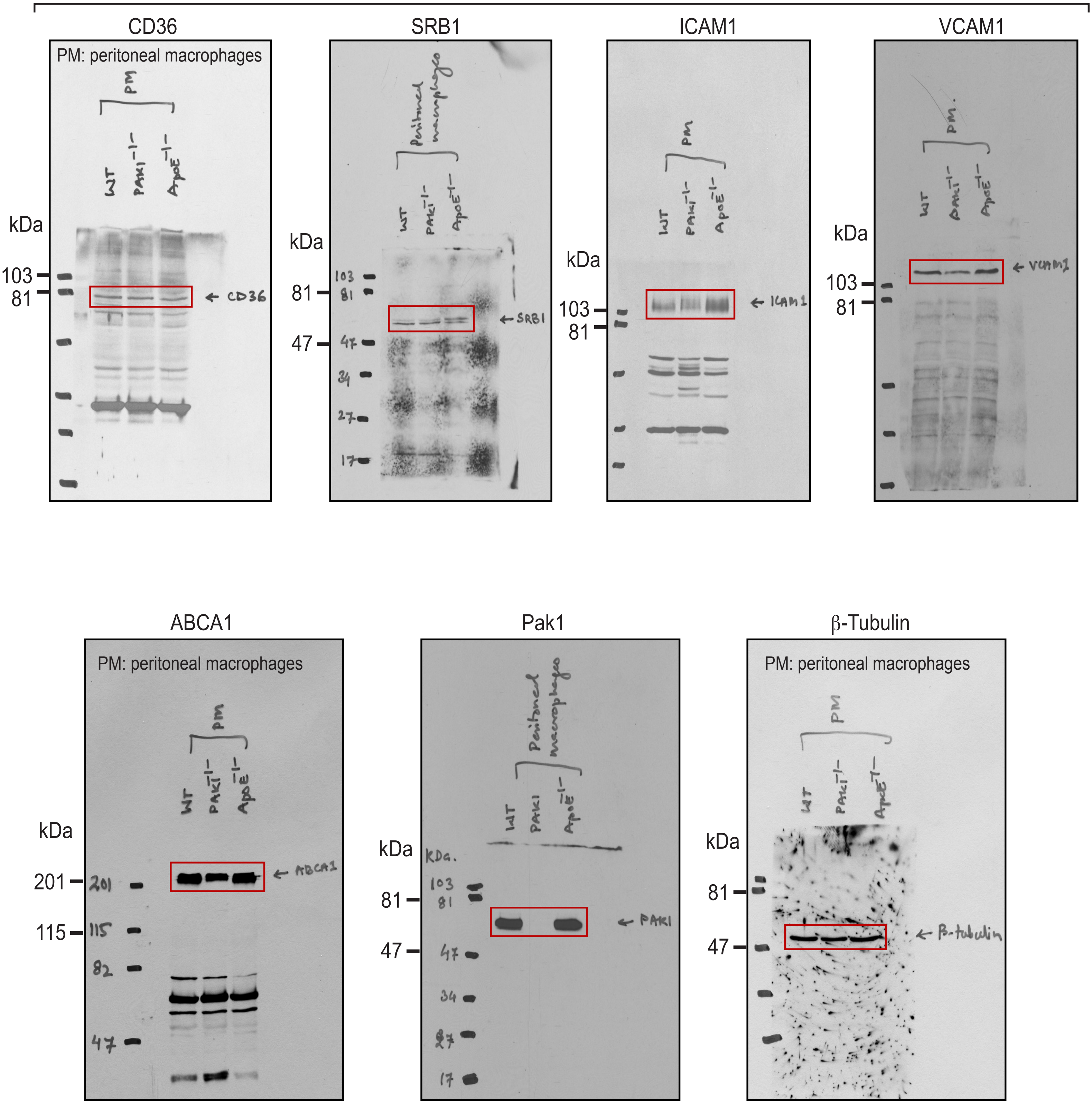

Supplementary Figure 6: Scans of immunoblots presented in Figure 4k.

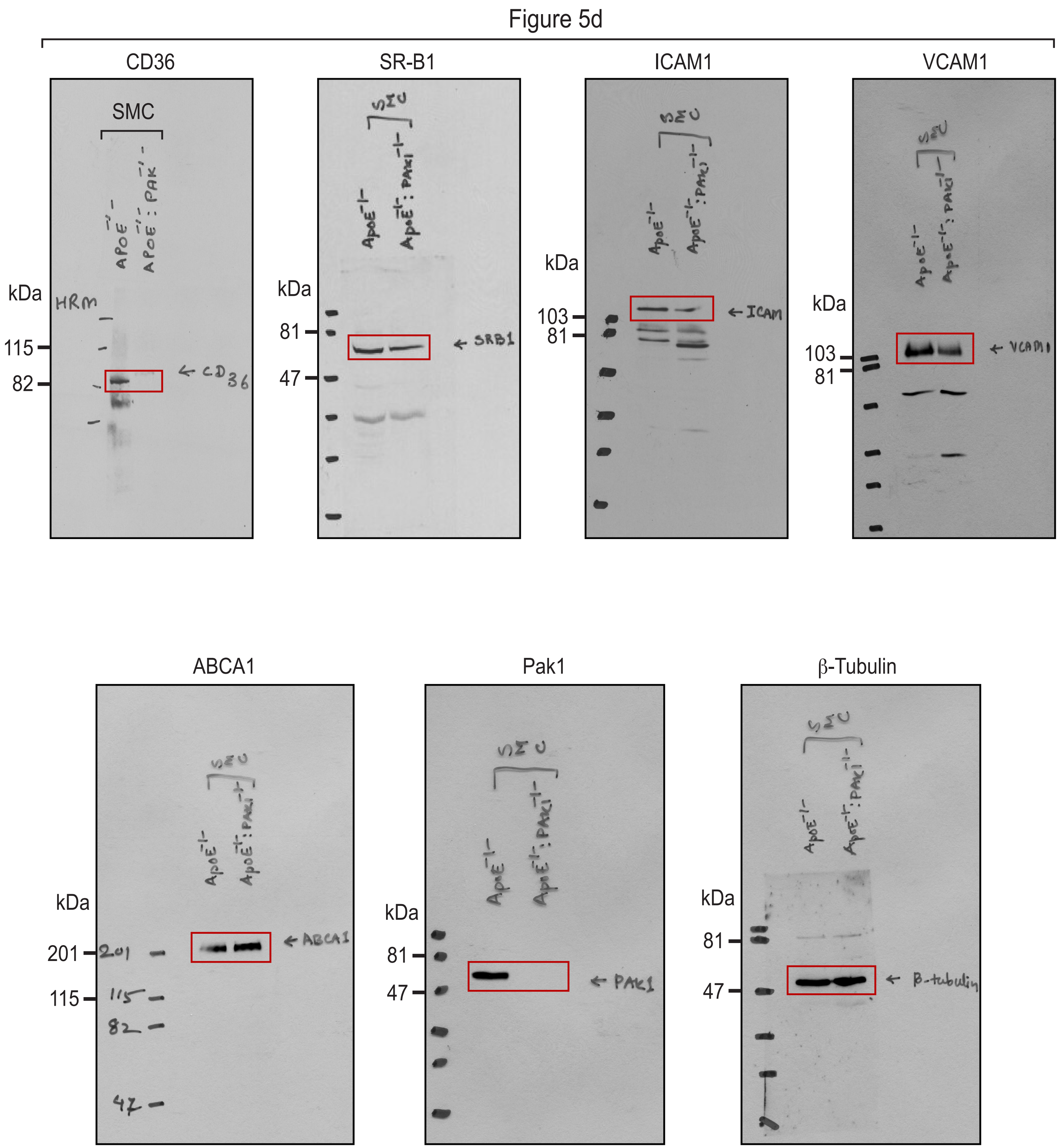

Supplementary Figure 7: Scans of immunoblots presented in Figure 5d.

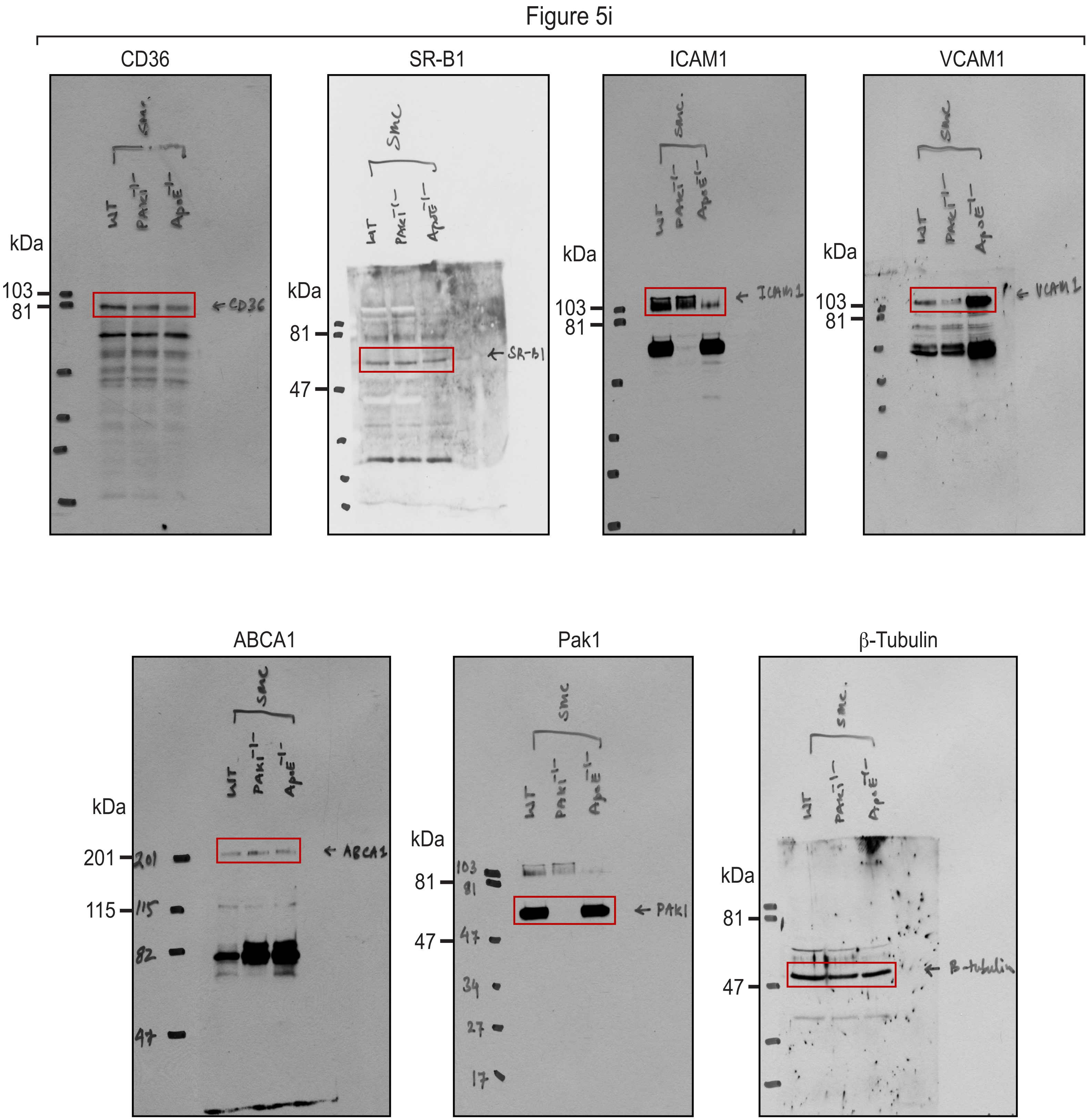

Supplementary Figure 8: Scans of immunoblots presented in Figure 5i.

Figure 6c

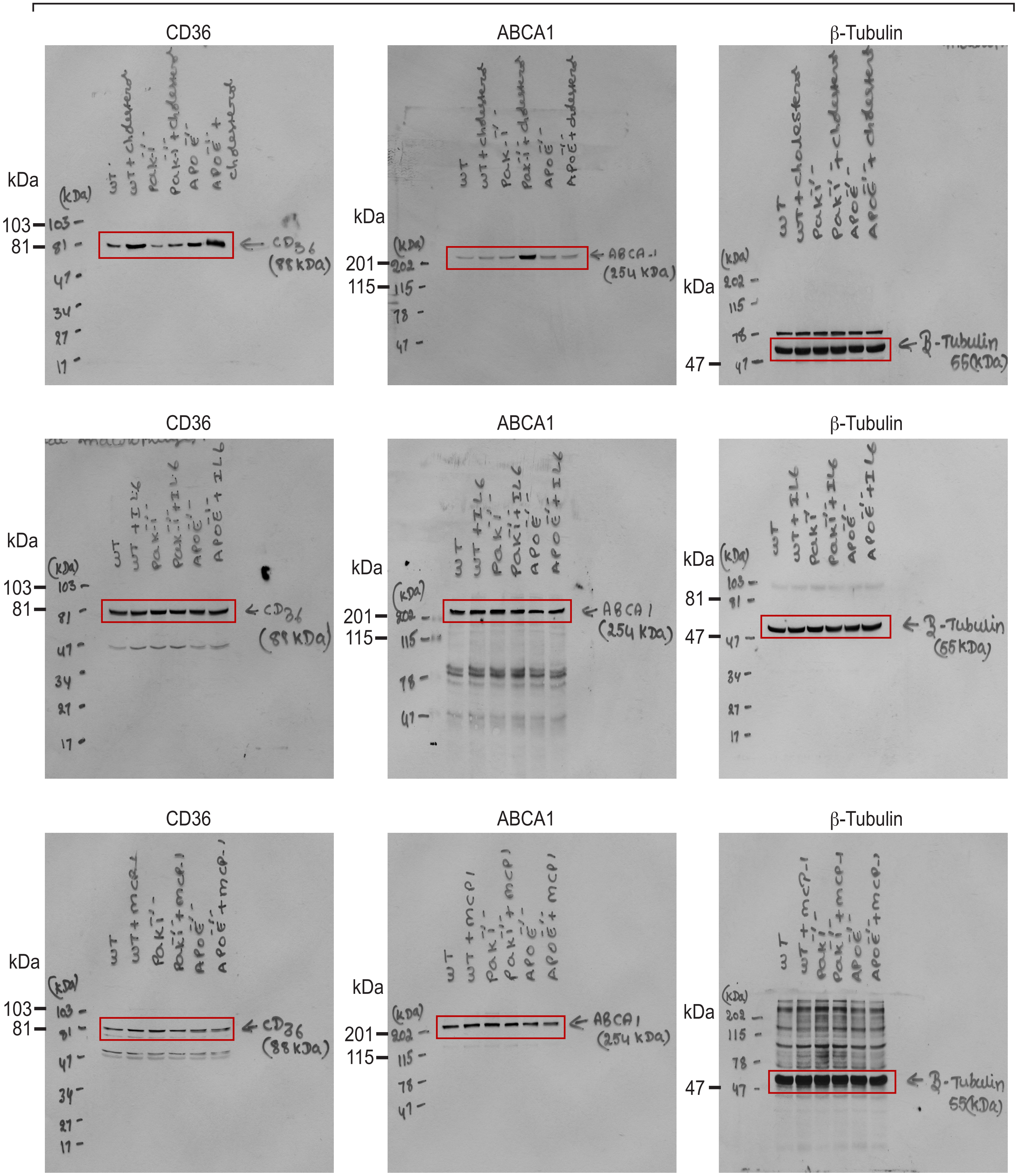

Supplementary Figure 9: Scans of immunoblots presented in Figure 6c.
